# CardioGenAI: a machine learning-based framework for re-engineering drugs for reduced hERG liability

**DOI:** 10.1186/s13321-025-00976-8

**Published:** 2025-03-05

**Authors:** Gregory W. Kyro, Matthew T. Martin, Eric D. Watt, Victor S. Batista

**Affiliations:** 1 Department of Chemistry, Yale University, New Haven, CT 06511 USA; 2 Drug Safety Research & Development, Pfizer Research & Development, Groton, CT 06340 USA

**Keywords:** Generative AI, Deep learning, Molecular optimization, hERG, Drug discovery, Machine learning, Transformers, Graph neural networks

## Abstract

The link between in vitro hERG ion channel inhibition and subsequent in vivo QT interval prolongation, a critical risk factor for the development of arrythmias such as Torsade de Pointes, is so well established that in vitro hERG activity alone is often sufficient to end the development of an otherwise promising drug candidate. It is therefore of tremendous interest to develop advanced methods for identifying hERG-active compounds in the early stages of drug development, as well as for proposing redesigned compounds with reduced hERG liability and preserved primary pharmacology. In this work, we present CardioGenAI, a machine learning-based framework for re-engineering both developmental and commercially available drugs for reduced hERG activity while preserving their pharmacological activity. The framework incorporates novel state-of-the-art discriminative models for predicting hERG channel activity, as well as activity against the voltage-gated Na_V_1.5 and Ca_V_1.2 channels due to their potential implications in modulating the arrhythmogenic potential induced by hERG channel blockade. We applied the complete framework to pimozide, an FDA-approved antipsychotic agent that demonstrates high affinity to the hERG channel, and generated 100 refined candidates. Remarkably, among the candidates is fluspirilene, a compound which is of the same class of drugs as pimozide (diphenylmethanes) and therefore has similar pharmacological activity, yet exhibits over 700-fold weaker binding to hERG. Furthermore, we demonstrated the framework's ability to optimize hERG, Na_V_1.5 and Ca_V_1.2 profiles of multiple FDA-approved compounds while maintaining the physicochemical nature of the original drugs. We envision that this method can effectively be applied to developmental compounds exhibiting hERG liabilities to provide a means of rescuing drug development programs that have stalled due to hERG-related safety concerns. Additionally, the discriminative models can also serve independently as effective components of virtual screening pipelines. We have made all of our software open-source at https://github.com/gregory-kyro/CardioGenAI to facilitate integration of the CardioGenAI framework for molecular hypothesis generation into drug discovery workflows.

**Scientific contribution**

This work introduces CardioGenAI, an open-source machine learning-based framework designed to re-engineer drugs for reduced hERG liability while preserving their pharmacological activity. The complete CardioGenAI framework can be applied to developmental compounds exhibiting hERG liabilities to provide a means of rescuing drug discovery programs facing hERG-related challenges. In addition, the framework incorporates novel state-of-the-art discriminative models for predicting hERG, Na_V_1.5 and Ca_V_1.2 channel activity, which can function independently as effective components of virtual screening pipelines.

**Supplementary Information:**

The online version contains supplementary material available at 10.1186/s13321-025-00976-8.

## Introduction

There is a well-established connection between in vitro blockade of the hERG (human Ether-à-go-go-Related Gene) potassium ion channel and in vivo QT interval prolongation, where the QT interval, as recorded on electrocardiograms, indicates the time between the start of the heart’s ventricular depolarization (i.e., the rapid influx of sodium ions that renders the cell’s interior less negatively charge) and the end of repolarization (i.e., the restoration of the cell’s membrane potential to its resting negative state) [1]. The hERG channel contributes to repolarization of the cardiac action potential by selectively allowing potassium ions to flow out of the cell following depolarization [2]. Inhibition of this channel can therefore directly disrupt cardiac repolarization, leading to prolongation of the QT interval, which consequently elevates the risk of potentially fatal arrythmias such as Torsade de Pointes (TdP) [3]. As a result, the potential propensity of drug candidates to present hERG liabilities is subject to rigorous regulatory scrutiny, and the pharmaceutical industry devotes a significant amount of resources to identifying hERG liabilities during early, preclinical and clinical phases of drug development [4].

The Comprehensive In Vitro Proarrhythmia Assay (CiPA) initiative [5], supported by regulatory agencies including the U.S. Food and Drug Administration (FDA), established guidelines for evaluating the proarrhythmia risk of drugs that also incorporate the voltage-gated sodium (Na_V_1.5) and calcium (Ca_V_1.2) ion channels alongside the hERG channel due to observations that modulating Na_V_1.5 and Ca_V_1.2 channel activities may mitigate the arrhythmogenic potential induced by hERG channel blockade [6–8]. A well-known example of this phenomenon is the case of verapamil, a drug that blocks both hERG and Ca_V_1.2 channels and is observed to have only a small impact on the QT interval, which is hypothesized to be due to the counteracting effects of Ca_V_1.2 blockade [9]. Additionally, Ca_V_1.2 blockade alone is reported to be a possible mechanism underlying undesirable blood-flow dynamics [10]. It is therefore of tremendous interest to develop highly capable methods for assessing how both prospective and currently available drugs interact with each of these three cardiac ion channels.

A multitude of experimental methods exist for in vitro determination of cardiac ion channel affinity [11–14]. However, they require synthesis of the compounds to be assayed, which is relatively time-consuming and expensive compared to in silico methods. Machine learning (ML)-based methods for predicting hERG channel activity have been extensively explored, utilizing both protein structure-based and ligand-based models [15–39]. However, structure-based predictive modeling of the hERG channel has proven to be difficult due to the channel’s intricate structure, its dynamic nature encompassing multiple conformations, and the possibility of unexpected interaction sites that are not apparent in conventional structural models [40]. For these reasons, ligand-based methods currently predominate. Predictive modeling for Na_V_1.5 and Ca_V_1.2 channel blocking is comparatively unexplored, as the amount of available data is much less compared to that for hERG. However, recent benchmarks for predicting Na_V_1.5 and Ca_V_1.2 channel activity have been established [41], and increasing effort is being devoted to developing models for these channels as well [42–45].

While ML-based discriminative models for predicting hERG channel activity have tremendous potential for applications in virtual screening, extending these capabilities to molecular generation through generative artificial intelligence (AI) can overcome the constraints of the currently available molecular libraries by enabling the direct in silico development of drugs with desired activities against cardiac ion channels. Numerous generative models have already demonstrated the ability to produce molecules with prespecified drug-like properties [46–105], and there has also been work aimed at generating molecules with desired on-target potency [53, 106, 107]. Despite the progress, there has been comparatively less effort devoted to developing and applying generative models for off-target potency optimization. Moreover, the abundance of available datapoints with low hERG activity, as opposed to the general scarcity of datapoints with high on-target potency for a given target, suggests that generative models for off-target potency optimization can more effectively identify patterns in the relevant chemical space and therefore be more successful than those for on-target potency optimization, further motivating method development in this area of research.

In this work, we present an ML-based framework designed to re-engineer both developmental and commercially available drugs for reduced hERG liability while retaining their pharmacological activity. The method utilizes a generative model to produce molecules conditioned on the molecular scaffold and physicochemical properties of the input hERG-active molecule. The generated ensemble is filtered using deep learning models for predicting hERG, Na_V_1.5 and Ca_V_1.2 channel activity. A chemical space representation is then constructed from the filtered generated distribution and the input molecule, where nearby molecules exhibit similar chemical properties, thus facilitating the identification of molecules with similar pharmacological activity to the input molecule but with reduced hERG channel inhibition. This approach, while not a replacement for the expertise of medicinal chemists, is highly effective at rapid molecular hypothesis generation, proposing refined candidates that can then be investigated with more expensive computational methods and experimental techniques.

## Overview of CardioGenAI framework

**Fig. 1:**
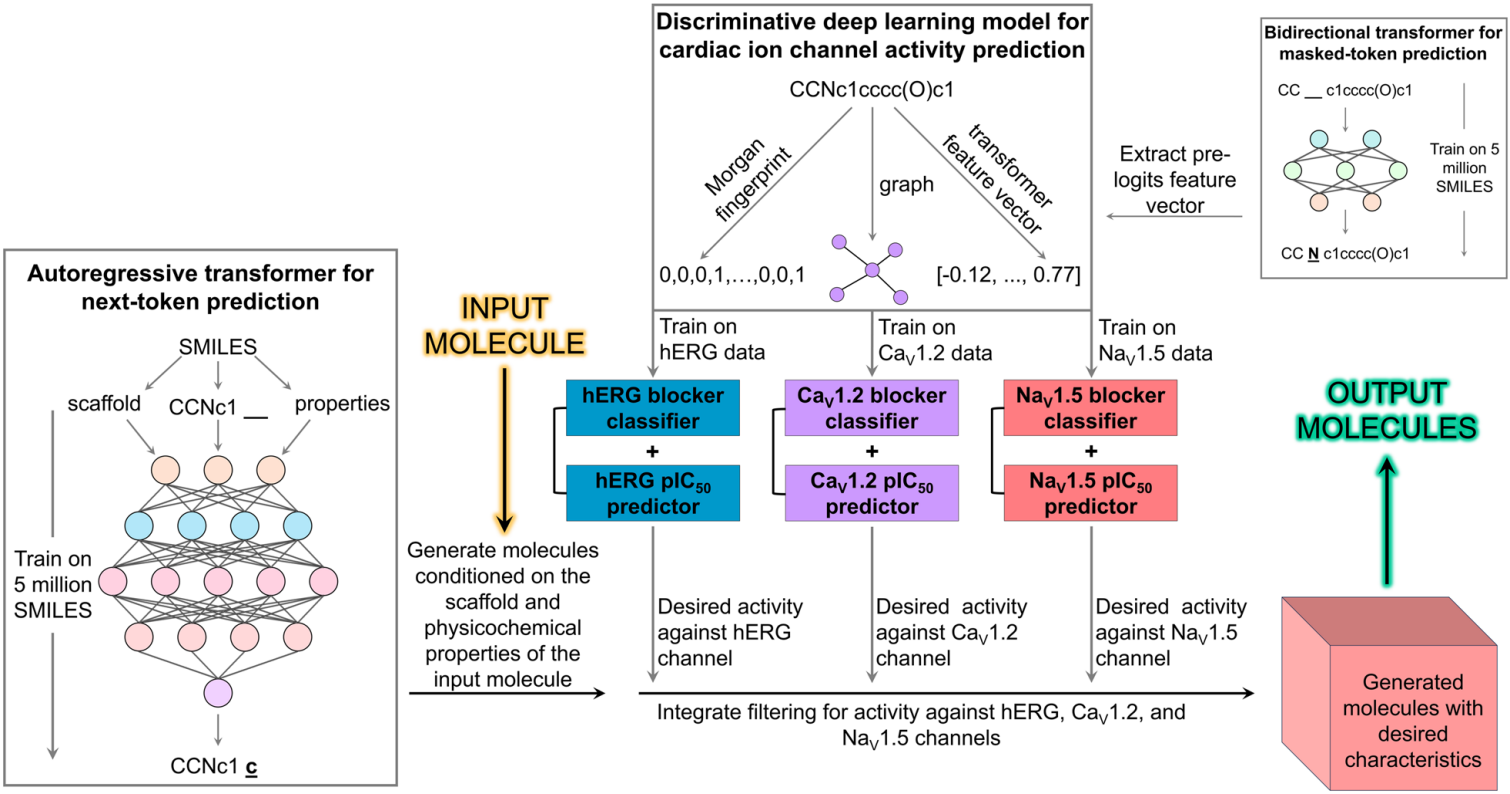
The CardioGenAI framework for re-engineering hERG-active compounds. An autoregressive transformer decoder pretrained on a large dataset of SMILES strings generates compounds conditioned on the scaffold and physicochemical properties of a given input compound, and the generated ensemble is filtered based on desired activity against hERG, Na_V_1.5 and Ca_V_1.2 channels. Cosine similarity is calculated between a 209-dimensional descriptor vector of the input compound and that of every filtered generated compound to identify the refined candidates most chemically similar to the input compound

The CardioGenAI framework combines generative and discriminative ML models to re-engineer hERG-active compounds for reduced hERG channel inhibition while preserving their pharmacological activity. A transformer decoder is trained on a dataset that we previously curated which contains approximately 5 million unique and valid SMILES strings derived from ChEMBL 33, GuacaMol v1, MOSES, and BindingDB datasets [108–112]. The model is trained autoregressively, receiving a sequence of SMILES tokens as context as well as the corresponding molecular scaffold and physicochemical properties, and iteratively predicting each subsequent token in the sequence. Once trained, this model, which is effectively a compression of the training set, is able to generate valid molecules conditioned on a specified molecular scaffold along with a set of physicochemical properties. For an input hERG-active compound, the generation is conditioned on the scaffold and physicochemical properties of this compound (Fig. 1A). Each generated compound is subject to filtering based on activity against hERG, Na_V_1.5 and Ca_V_1.2 channels. Depending on the desired activity against each channel, the framework employs either classification models to include predicted non-blockers (i.e., pIC_50_ value ≤ 5.0) or regression models to include compounds within a specified range of predicted pIC_50_ values. Both the classification and regression models utilize the same architecture, and are trained using three feature representations of each molecule: a feature vector that is extracted from a bidirectional transformer trained on SMILES strings, a molecular fingerprint, and a graph (more details in Sect. "Data Featurization"). For each molecule in the filtered generated ensemble and the input hERG-active molecule, a feature vector is constructed from the 209 2D chemical descriptors available through the RDKit Descriptors module [113]. The redundant descriptors are then removed according to pairwise mutual information calculated for every possible pair of descriptors. Cosine similarity is then calculated between the processed descriptor vector of the input molecule and the descriptor vectors of every filtered generated molecule to identify the refined candidates most chemically similar to the input molecule (Fig. 1B).

## Discriminative models for predicting cardiac ion channel activity

### Data featurization

For training and evaluation of hERG, Na_V_1.5 and Ca_V_1.2 inhibition prediction models, we utilize the training and evaluation datasets included in the benchmarks recently developed by Arab et al. [41] These benchmarks are designed to assess model generalizability, enforcing a maximum fingerprint similarity cutoff between molecules in the training and evaluation sets. Multiple published models in the field have been assessed using evaluation sets that have significant overlap with the corresponding training sets [38, 114], undoubtedly yielding overoptimistic results with respect to the models’ abilities to generalize. The compounds in the evaluation sets used in this work have a structural similarity, as determined by pairwise Tanimoto similarity between 2048-bit Morgan fingerprints, no greater than 0.70 to any compound in the corresponding training or validation sets. Compounds were sourced from the ChEMBL bioactivity database [115–117], PubChem [118], BindingDB [112, 119], hERGCentral [120], and the scientific literature [38, 121–123]. Each molecule is represented as a SMILES string which was canonicalized using RDKit, and labeled with the experimentally determined cardiac ion channel pIC_50_ value. For compounds with multiple experimentally determined pIC_50_ values, the assigned label is calculated as the mean value while retaining only those within the 95th percentile to minimize the influence of outliers. For binary classification tasks, compounds with a pIC_50_ value greater than or equal to 5.0 are labeled as blockers. For hERG, Na_V_1.5 and Ca_V_1.2 channels, training sets contain 17 796 (78.3%), 1 653 (74.8%), and 641 (72.6%) datapoints, validation sets contain 4 450 (19.6%), 414 (18.7%), and 161 (18.2%) datapoints, and test sets contain 474 (2.1%), 142 (6.4%), and 81 (9.2%) datapoints, respectively. For more details regarding the curation of the datasets, we refer readers to the original paper. [41]

It is important to note that variations in experimental protocols could contribute to discrepancies in measured pIC_50_ values for each channel due to differences in the probabilities of each channel occupying open, closed and inactivated states [124, 125]. Moreover, it has been demonstrated that systematic differences in assay conditions, such as temperature, voltage protocols, and buffer composition, can lead to significant discrepancies in reported values. For instance, even minor deviations in experimental setup have been shown to cause variability exceeding 0.5 log units in pIC_50_ values for the same compound across different studies [126]. Thus, given that the datasets used are curations of publicly available data that were obtained via different experimental protocols, variability in the experimental conditions and state probabilities may set an artificial limit on the predictive accuracy that models can achieve.

We found there to be a positive correlation (Pearson r = 0.256) between hERG pIC_50_ values and the logarithm of the partition coefficient between n-octanol and water (LogP), as well as a negative correlation (Pearson r = -0.215) with topological polar surface area (TPSA) (Figure S1 in Additional file 1). These findings are consistent with established medicinal chemistry knowledge that increasing polarity or reducing lipophilicity reduces hERG channel blockade [127]. Additionally, we also identified a relation between hERG pIC_50_ values and the presence of charged nitrogen atoms within aromatic or hydrophobic groups among the molecules exhibiting the most substantial hERG activity (Figure S2 in Additional file 1).

**Fig. 2:**
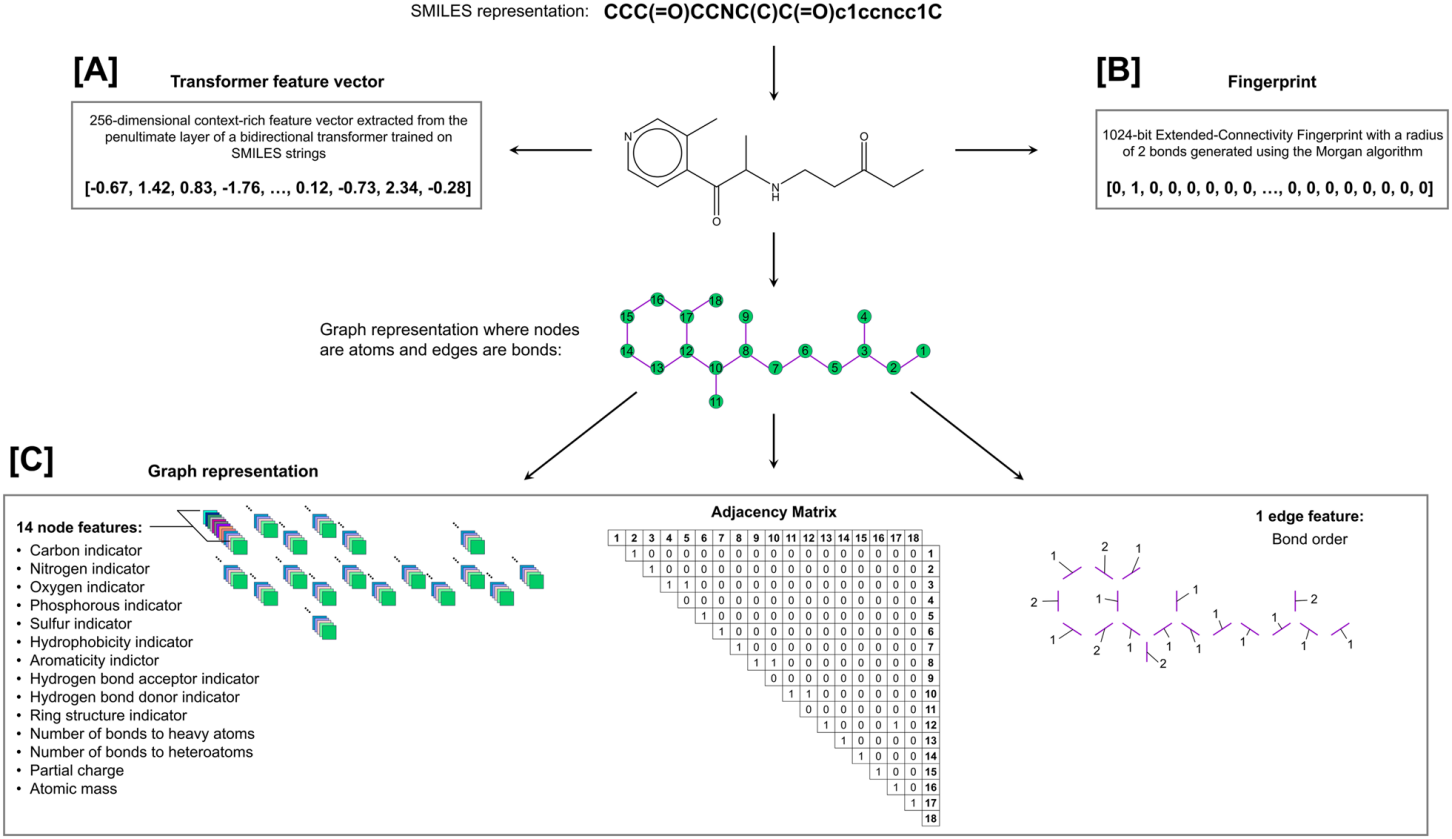
Featurization of a SMILES string—CCC(= O)CCNC(C)C(= O)c1ccncc1C—for use by the CardioGenAI discriminative models. The SMILES string is represented as **A** a 256-dimensional feature vector that is extracted from the penultimate layer of a bidirectional transformer trained on SMILES strings, **B** a 1024-bit Extended-Connectivity Fingerprint with a diameter of 4 bonds (ECFP4) generated using the Morgan algorithm, and **C** a graph

We represent each compound as three distinct forms: a 256-dimensional feature vector that is extracted from a bidirectional transformer trained on SMILES strings, a 1024-bit Extended-Connectivity Fingerprint with a diameter of 4 bonds (ECFP4) generated using the Morgan algorithm, and a graph (Fig. 2). A bidirectional transformer is first trained for masked-token prediction on the same dataset used to train the autoregressive transformer, allowing it to develop an intricate internal representation of molecular structure and grasp the syntax of SMILES notation (more details in Sect. "Data Preparation"). After this model is fully trained, it is used as a means of extracting a context-rich feature vector as a representation of a given SMILES string. Specifically, we extract the processed vector from the penultimate layer of the model corresponding to the start token, which contains information about the entire SMILES string that contributes to the prediction of a masked token within the sequence. This information encapsulates nuanced inter-token relationships and patterns among different molecules, rendering this feature vector a powerful representation that captures important characteristics of the molecule in a high-dimensional space (more details in Sect. "Model Architectures").

In the graph representation, nodes are atoms and edges are bonds. Each node is represented as a 14-dimensional vector of atomic features: carbon indicator, nitrogen indicator, oxygen indicator, phosphorous indicator, sulfur indicator, hydrophobicity indicator, aromaticity indicator, hydrogen bond acceptor indicator, hydrogen bond donor indicator, ring structure indicator, number of bonds to heavy atoms, number of bonds to heteroatoms, partial charge, and atomic mass. Each edge is labeled with the corresponding bond order.

### Model Architecture

**Fig. 3:**
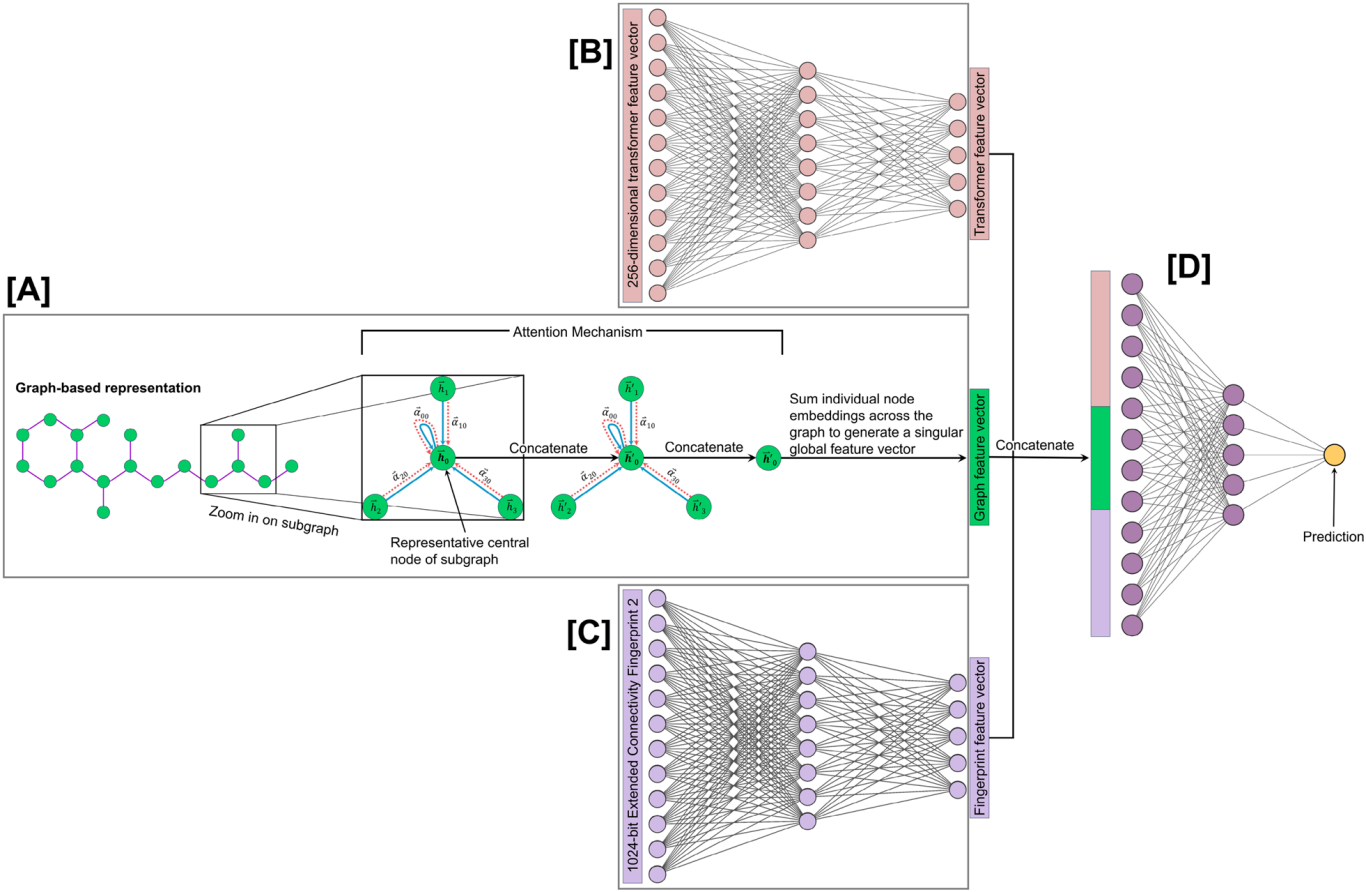
Illustration of the forward pass of the CardioGenAI discriminative models. The graph representation of a given SMILES string is encoded by **A** a graph attention network (GAT). The **B** transformer-derived and **C** fingerprint feature vectors are encoded by feed-forward networks. These three encodings are then concatenated and passed to **D** a final feed-forward network to generate a prediction

The transformer-based feature vector and the ECFP4 are each processed by separate two-layer feed-forward networks (Fig. 3B, C). For each of the two layers of the networks, the input vector undergoes a linear transformation followed by batch normalization. The normalized output is then passed through a ReLU activation function, followed by dropout with a rate of 50%.

The graph representation is processed by a graph attention network (GAT) consisting of two GAT convolutional layers (Fig. 3A). Initially, the graph is augmented with self-loops to ensure that each node’s feature vector is included in its own neighborhood during feature aggregation. The fist GAT layer transforms the node feature vectors through a linear operation, followed by a softmax-based attention mechanism to assign weights to the features of each node’s neighbors, relative to the source node. The output of this layer is passed through a ReLU activation function and fed to the second GAT convolutional layer which operates analogously to the first layer. After being processed by the second GAT convolutional layer, the updated node features are aggregated to form a graph-level representation using a global add pooling operation, which sums the node features across all nodes to generate a single vector that encapsulates the entire graph’s information.

After each of the three input feature representations has been encoded, they are concatenated to form a combined feature vector. This combined feature vector is then passed through a two-layer feed-forward network (Fig. 3D). The first layer applies a linear transformation to the feature vector followed by batch normalization. The normalized output is then passed through a ReLU activation function followed by dropout with a rate of 50%. The output of this layer then undergoes a linear transformation to map it to the final output space.

### Trainings and hyperparameters

The classification and regression models for each cardiac ion channel were trained for 200 and 100 epochs, respectively, with a batch size of 32; we trained the classification models for an additional 100 epochs because the training loss had not converged after only 100 epochs (Figure S3 of Additional File 1). The AdamW optimizer, a variant of the Adam optimizer that incorporates weight decay for regularization, was used with a learning rate of 3 × 10^–4^ and a weight decay of 1 × 10^–4^ to optimize the models’ parameters. Additionally, L1 regularization was applied with a regularization coefficient of 1 × 10^–4^ to induce sparsity within the model parameters. We integrated a learning rate scheduler which monitors the validation loss and halves the learning rate if no improvement is observed for 10 consecutive epochs. To ensure stability in training and prevent gradient explosion, gradient clipping was applied with a maximum norm of 5.0. For the classification and regression models, binary cross entropy loss and mean squared error loss were used as objective functions, respectively. The model parameters used for inference are those from the epoch with the highest validation accuracy for classification and highest validation Pearson correlation for regression. Learning curves for each of the classification and regression models are reported in Figure S3 of Additional file 1.

### Benchmarking against existing models

We found that utilizing all three feature representations (i.e., transformer-based feature vector, fingerprint, and graph) achieves the best performance on the hERG blocker classification benchmark compared to using any other possible combination of feature representations (Table S4 in Additional file 1), and we therefore adopt this combination of feature representations for our classification models.

We compare the performance of our classification models to the highest-performing models in the literature that have been evaluated with the benchmarks used in this work. Computed metrics include:

1 $$\text{Accuracy }(\text{AC})=\frac{TP+TN}{TP+TN+FP+FN}$$

2 $$\text{Sensitivity }(\text{SN})=\frac{TP}{TP+FN}$$

3 $$\text{Specificity }(\text{SP})=\frac{TN}{TN+FP}$$

4 $$\text{F}1-\text{score }(\text{F}1)=\frac{TP}{TP+\frac{1}{2}\left(FP+FN\right)}$$

5 $$\text{Correct Classification Rate }(\text{CCR})=\frac{SN+SP}{2}$$

6 $$\text{Matthews Correlation Coefficient }(\text{MCC})=\frac{TP\times TN-FP\times FN}{\sqrt{\left(TP+FP\right)\times \left(TP+FN\right)\times \left(TN+FP\right)\times \left(TN+FN\right)}}$$

where $TP$, $TN$, $FP$, and $FN$ represent the number of true positives, true negatives, false positives, and false negatives, respectively. We find that our hERG blocker classification model outperforms all existing models in the literature on the hERG benchmark for binary classification (Table 1).

**Table 1:** Performance of CardioGenAI for binary classification of hERG blockers compared to that of the highest-performing models in the literature on the benchmark created by Arab et al. [41]

Model	AC	SN	SP	F1	CCR	MCC
CardioGenAI	**83.5**	86.2	**80.3**	**85.1**	**83.2**	**66.7**
CToxPred-hERG	81.4	**86.7**	74.6	83.9	80.7	62.1
CardioTox	81.2	83.0	78.9	83.1	81.0	61.9
ADMETlab 2.0	71.7	71.6	71.8	73.8	71.7	43.1
ADMETsar 2.0	68.5	84.5	48.3	75.0	66.4	35.5
CardPred	56.1	52.7	60.3	57.0	56.5	13.0

^a^Compounds in the test set have a structural similarity, as determined by pairwise Tanimoto similarity between 2048-bit Morgan fingerprints, no greater than 0.70 to any compound in the corresponding training or validation sets

^b^The top value achieved for each metric is shown in bold

^c^Accuracy (AC), sensitivity (SN), specificity (SP), F1-score (F1), correct classification rate (CCR), and Matthew’s correlation coefficient (MCC) are shown

^d^Results are shown for CToxPred-hERG [41], CardioTox [33], ADMETlab 2.0 [128], ADMETsar 2.0 [129], and CardPred. [21]

The improvement of our hERG blocker predictive model over previous models justifies its use within the CardioGenAI framework as opposed to other predictive models which have already been developed.

For the Na_V_1.5 and Ca_V_1.2 benchmarks, only the models presented by Arab et al. [41] have been evaluated, largely owing to the fact that these benchmarks have only recently been developed and the experimental data available for these channels is scarce compared to that for hERG. We find that our models demonstrate superior performance for both Na_V_1.5 and Ca_V_1.2 channels (Table 2). Additionally, the area under the curve (AUC) of the receiver operating characteristic for each channel is commensurate with the accuracy that our models obtain; hERG AUC is 0.88, Na_V_1.5 AUC is 0.89, and Ca_V_1.2 AUC is 0.95 (Figure S5B in Additional file 1).

**Table 2:** Performance of CardioGenAI for binary classification of Na_V_1.5 and Ca_V_1.2 blockers compared to that of the models created by Arab et al. [41]

Channel	Model	AC	SN	SP	F1	CCR	MCC
Na_V_1.5	CardioGenAI	**89.4**	**95.9**	**75.6**	**92.5**	**85.7**	**75.1**
	CToxPred-Nav	81.7	85.6	73.3	86.5	79.5	58.2
Ca_V_1.2	CardioGenAI	**91.4**	**96.2**	**82.8**	**93.5**	**89.5**	**81.0**
	CToxPred-Cav	86.4	**96.2**	69.0	90.1	82.6	70.2

^a^Compounds in the test set have a structural similarity, as determined by pairwise Tanimoto similarity between 2048-bit Morgan fingerprints, no greater than 0.70 to any compound in the corresponding training or validation sets

^b^The top value achieved for each metric is shown in bold

^c^Accuracy (AC), sensitivity (SN), specificity (SP), F1-score (F1), correct classification rate (CCR), and Matthew’s correlation coefficient (MCC) are shown

We report the performance of our regression models in Figure S5C-E and Table S6 in Additional file 1. The Pearson correlation between true pIC_50_ values and those predicted by our regression models are 0.67 for hERG, 0.60 for Na_V_1.5, and 0.81 for Ca_V_1.2 benchmarks (Figure S5C-E in Additional file 1).

In order to provide interpretability of the regression models’ predictions, we calculate the correlation between predicted pIC_50_ values and each property in a set of physicochemical properties for each of the three cardiac ion channels (Table S7 in Additional file 1). The key findings of this analysis are as follows: predicted hERG pIC_50_ values correlate positively with the number of rotatable bonds (Pearson r = 0.327) and LogP (r = 0.321); predicted Na_V_1.5 pIC_50_ values correlate negatively with the number of hydrogen bond donors (r = − 0.593) and TPSA (r = − 0.545), while correlating positively with LogP (r = 0.406); and predicted Ca_V_1.2 pIC_50_ values correlate positively with the number of hydrogen bond acceptors (r = 0.621), TPSA (r = 0.581), the number of heteroatoms (r = 0.555), molecular weight (r = 0.444) and the number of rotatable bonds (r = 0.318), while correlating negatively with the number of rings (r = − 0.315).

Additionally, in order to ensure that the predictive abilities of our models are not artifacts of spurious correlations within the data, we perform Y-randomization tests for all discriminative models and report results in Table S8 and Figure S9 of Additional file 1.

### Application to the drugcentral database of FDA-approved drugs

To demonstrate the practical utility of our classification and regression models, we applied them to the FDA-approved drugs from the DrugCentral database, offering a real-world context for assessing cardiac ion channel inhibition [130, 131]. It is important to note that many of the compounds occur in the training set of the discriminative models. Thus, predictive ability for these compounds should not be interpreted as validation of the models’ predictive ability for unseen compounds. Of the 1692 unique FDA-approved drugs, we classify 504 (29.8%) to be hERG blockers (i.e., pIC_50_ value ≥ 5.0), 764 (45.2%) to be Na_V_1.5 blockers, and 400 (23.6%) to be Ca_V_1.2 blockers (Figure S10A in Additional file 1). A more complete analysis of the predicted cardiac ion channel activity of the FDA-approved drugs is reported in Figure S10B of Additional file 1. In addition, we report the compounds with a predicted hERG pIC_50_ value above 7.0 (i.e., more than 100-fold greater hERG inhibitory potency than the blocker threshold) in Table 3.

**Table 3:** Analysis of the FDA-approved compounds from the DrugCentral database with a predicted hERG pIC_50_ value above 7.0

Drug Name	Pharmacological Indication	Mechanism of Action	FDA Approval Status	Predicted hERG pIC_50_	In Training Set	Experimentally Determined hERG pIC_50_
Nintedanib	Idiopathic pulmonary fibrosis	Kinase inhibitor	Approved	8.234	yes	8.585
Ibutilide	Atrial fibrillation, atrial flutter	hERG channel blocker	Approved	7.977	yes	8.000
Pimozide	Tourette’s disorder	Dopamine D2 receptor antagonist	Approved	7.629	yes	8.520
Halofantrine	Severe malaria	Forms toxic complexes with ferritoporphyrin IX	Approved	7.588	no	7.398
Astemizole	Allergy symptoms	H_1_-receptor antagonist	Withdrawn due to concerns of arrhythmias	7.562	yes	8.538
Tolterodine	Overactive bladder	Muscarinic receptor antagonist	Approved	7.311	no	7.886
Droperidol	Nausea and vomiting in surgical and diagnostic procedures	Dopamine D2 receptor antagonist	Approved	7.300	yes	7.495
Dofetilide	Atrial fibrillation, atrial flutter	hERG channel blocker	Approved	7.164	yes	8.194
Haloperidol decanoate	Schizophrenia, psychotic disorders, Tourette’s disorder	Dopamine D2 receptor antagonist	Approved	7.149	no	6.921
Amiodarone	Recurrent ventricular fibrillation, recurrent hemodynamically unstable ventricular tachycardia	hERG channel blocker	Approved	7.127	yes	7.523
Terfenadine	Allergic rhinitis, hay fever, allergic skin disorders	H_1_-receptor antagonist	Withdrawn due to concerns of arrhythmias	7.022	yes	7.252

^a^Information regarding the pharmacological indication, mechanism of action, and FDA approval status for each drug is obtained from DrugBank [132, 133]

^b^Experimentally determined hERG pIC_50_ values are obtained from the hERG dataset curated by Arab et al. [41]

For the 11 FDA-approved compounds with a predicted hERG pIC_50_ value greater than 7.0, the predicted pIC_50_ values are closely aligned with those that are experimentally determined, with notable agreement in cases where the compound is not in the training set of the model (Table 3). However, for three of the compounds, namely pimozide, astemizole, and dofetilide, each predicted hERG pIC_50_ value differs from the corresponding experimentally determined value by about an order of magnitude. The experimentally determined pIC_50_ values for these three compounds are among the top four highest values in the set of FDA-approved compounds, and each is greater than three standard deviations above the mean pIC_50_ value in the training distribution. Because these high values are not well-represented in the training set, the model’s tendency to regress toward the mean pIC_50_ value likely accounts for the observed discrepancy between predicted and experimentally determined pIC_50_ values for these three compounds (see Figure S5C in Additional File 1).

The primary mechanism of action for three of the 11 drugs is to block the hERG channel: ibutilide [134], dofetilide [135], and amiodarone [136]. Another three of them function primarily as dopamine D2 receptor antagonists: pimozide [137], droperidol [138], and haloperidol decanoate [139]. Pimozide is reported to cause QT interval prolongation and ventricular arrhythmias due to hERG channel blockade with high specificity and affinity [140]; droperidol is reported to cause TdP due to potent hERG channel blockade [141]; haloperidol decanoate has been found to cause sudden death due to hERG channel blockade-induced QT interval prolongation. [142]

Another two of the 11 drugs function primarily as H_1_-receptor antagonists: astemizole and terfenadine [143, 144]. Both of these drugs were withdrawn from the market due to hERG blockade-induced cardiac arrhythmias [145, 146]. Of the remaining three drugs of the 11, nintedanib is reported to cause side effects related to hERG channel blockade [147], halofantrine is found to cause hERG blockade-induced QT interval prolongation [148], and tolterodine is reported to cause hERG blockade-induced tachycardia and palpitations [149]. These results support the real-world application of CardioGenAI to hERG activity prediction.

### Limitations of the discriminative models

While the discriminative models used in the CardioGenAI framework demonstrate robust predictive performance, certain limitations should be acknowledged. A key limitation arises from the variability in the experimental protocols used to obtain pIC_50_ labels. These protocols often differ in assay conditions, measurement methodologies, and the probabilities of cardiac ion channels occupying open, closed, or inactivated states. Such variability introduces noise into the data and may impose an artificial upper bound on the predictive accuracy achievable by models trained on publicly available hERG data.

Additionally, the models’ performance is likely influenced by the inherent biases present in the training data. For example, underrepresentation of certain chemical scaffolds or activity ranges could impact the generalizability of the models to the corresponding regions of chemical space.

## Transformer-based models

### Data preparation

The generative autoregressive transformer and the bidirectional transformer used for extracting features to be utilized by the discriminative models are both trained with a dataset that we previously curated by combining all of the unique and valid SMILES strings from ChEMBL 33, GuacaMol v1, MOSES, and BindingDB datasets [108–112]. The combined dataset initially had a vocabulary of 196 unique tokens. To reduce the size of the vocabulary and thus improve the computational efficiency of the transformer models, we removed all SMILES strings containing at least one token that appeared less than 1 000 times in the combined dataset; most of the SMILES strings that were excluded contain rare transition metals or isotopes. Of the remaining SMILES strings, the longest one contained 1 503 tokens, while 99.99% of the strings in the entire remaining dataset had 133 or fewer tokens. In order to reduce the block size of our transformer models, and thus further improve the computational efficiency, we removed all SMILES strings from the dataset that contained more than 133 tokens. The remaining SMILES strings were then extended, if necessary, to a length of 133 using a padding token “ < pad > ”, and augmented with a start token “[CLS]” and an end token “[EOS]”. The processed dataset contains approximately 5.5 million SMILES strings which are randomly split into training (5 262 776 entries; 95%) and validation (276 989 entries; 5%) sets. Please refer to our previous paper for complete details regarding SMILES string preprocessing. [108]

For each SMILES string, we calculated the molecular scaffold using the Murcko algorithm [150], which identifies the core structure by removing side chains from the molecular graph, retaining the ring systems and the linkers connecting them. We also calculated ten physicochemical properties for each SMILES string: molecular weight, number of rings, number of rotatable bonds, number of hydrogen bond donors, number of hydrogen bond acceptors, TPSA, number of heteroatoms, LogP, number of stereocenters, and formal charge.

### Model architectures

For a given SMILES string, the autoregressive transformer considers the sequence of the SMILES string, the molecular scaffold, and the set of physicochemical properties, while the bidirectional transformer only considers the sequence. For both models, tokens in the sequence are embedded using a learnable embedding table, where each token in the vocabulary corresponds to a learnable embedding vector. The positions of the tokens in the sequence are embedded using a separate learnable embedding table, where each index in the sequence corresponds to a learnable embedding vector that allows the model to account for a given token’s position in the sequence and capture sequential context within the SMILES string. For the autoregressive transformer, the set of physicochemical properties is mapped to the embedding dimension via a learnable linear transformation, and the molecular scaffold is embedded using a learnable embedding table analogous to that used for the token embeddings. For both models, all embeddings, each with an embedding dimension of 256, are summed to create a combined feature representation, and then dropout is applied with a rate of 10%.

The transformer architecture used consists of eight sequential blocks, each beginning with layer normalization to stabilize the input. This is followed by a self-attention mechanism, where query $\left(Q\right)$, key $\left(K\right)$, and value $\left(V\right)$ vectors are computed for each input token, attention scores are derived via a scaled dot product of $Q$ and $K$ vectors, and the softmax function normalizes these scores to obtain weights that modulate the aggregation of $V$, effectively capturing the magnitude with which each token will attend to every other token in the sequence. The self-attention mechanism is executed multiple times in parallel through what is referred to as multi-head attention. The models used in this work employ eight attention heads, where each head uses its own set of learned linear transformations to generate $Q$, $K$, and $V$ vectors for each token in the sequence, allowing the model to simultaneously focus on different aspects of the input across the various heads. Representative attention maps for the autoregressive and bidirectional transformers are reported in Figures S11 and S12 of Additional file 1.

The outputs of all attention heads are concatenated and passed through a learned linear transformation to generate the final output of the multi-head attention mechanism. A residual connection then merges this output with the initial block input. The resulting data tensor then undergoes another layer normalization and progresses through a two-layer feed-forward network with a 10% dropout rate and GeLU activation, before reintegration with its pre-normalized state. The final step involves another layer normalization, followed by a linear transformation that projects the data tensor onto the vocabulary space, generating a logits vector (i.e., the unnormalized log probabilities for each token in the vocabulary). When using the trained bidirectional transformer to derive feature vectors to be utilized by the discriminative models, the data tensor is extracted immediately prior to the final linear transformation, and the vector corresponding to the start token is used as the feature vector.

### Trainings and hyperparameters

The autoregressive transformer is trained for next-token prediction, and the bidirectional transformer is trained for masked-token prediction where each token in a given SMILES sequence has a 15% probability of being selected; of these, 80% are replaced with a mask token “ < MASK > ”, 10% are replaced with a random token from the vocabulary, and the remaining 10% are left unchanged. Both models were trained for 100 epochs with a batch size of 512. The Sophia optimizer was used with a learning rate of 3 × 10^–4^ and a weight decay of 1 × 10^–1^, [151] and cross entropy loss was used as the objective function for both models. The model parameters used for inference are those from the last epoch of training. Learning curves for the autoregressive and bidirectional transformers are reported in Figure S13 of Additional file 1.

### Molecular generation

The autoregressive transformer is used to generate SMILES strings, conditioned on both a molecular scaffold and a set of ten physicochemical properties. To rigorously evaluate the model’s ability to generate molecules with prespecified physicochemical properties, we fix one property at a time to a discrete value while the other nine properties are sampled using a random uniform distribution within ranges of acceptable values based on ADMETlab 2.0 guidelines for medicinal chemistry [128]. This procedure is performed for 500 molecules per fixed property value. For example, we generate 500 molecules conditioned on a molecular weight of 400 $\frac{g}{mol}$ and another 500 conditioned on a molecular weight of 600 $\frac{g}{mol}$ to assess the model’s ability to generate molecules with a targeted molecular weight. We repeat this approach for each physicochemical property, and observe that the model is able to successfully generate molecular distributions that satisfy the prespecified criteria (Figure S14A-I in Additional file 1). We also demonstrate the model’s ability to generate molecules conditioned on multiple discrete physicochemical property values simultaneously (e.g., TPSA of 50 Å [2] and molecular weight of 350 $\frac{g}{mol}$), validating its utility and justifying its use within the CardioGenAI framework (Figure S14J in Additional file 1).

## Complete CardioGenAI framework

### High-level description of the workflow

The fundamental objective of the CardioGenAI framework is to re-engineer hERG-active compounds for reduced hERG activity while preserving their pharmacological action. Within the framework, the autoregressive transformer first generates valid molecules conditioned on the molecular scaffold and physicochemical properties of the input hERG-active molecule, which are filtered based on desired activity against hERG, Na_V_1.5 and Ca_V_1.2 channels using the discriminative models. The input molecule and each filtered generated molecule are then converted into 209-dimensional chemical descriptor vectors which are refined by removing the redundant descriptors according to pairwise mutual information between every possible descriptor pair. Cosine similarity is then calculated between the descriptor vector of the input molecule and the descriptor vectors of every filtered generated molecule to identify the molecules most chemically similar to the input molecule but with desired activity against each of the cardiac ion channels.

### Case study: optimizing the FDA-approved drug pimozide for reduced hERG activity

Pimozide is an FDA-approved antipsychotic agent that is used to treat Tourette’s syndrome as well as various other psychiatric disorders [152]. Its main pharmacodynamic action is to blockade dopamine D2 receptors on neurons in the central nervous system (CNS); it also has various effects on other CNS receptor systems which are not fully characterized [137]. There are many reports linking the use of pimozide to QT interval prolongation and ventricular arrythmias [153, 154], and there are multiple reported instances of sudden, unexpected deaths of patients receiving pimozide [155].

It was initially observed clinically that only a very low dose of pimozide is necessary to produce QT interval prolongation, suggesting that it binds to one or more cardiac potassium ion channels with high affinity [153]. Subsequent experimental validation indicated pimozide’s high affinity to the hERG channel, evidenced by its potent inhibitory effect with an IC_50_ value of approximately 18 nM [140].

Because of pimozide’s proarrhythmic effects, it is contraindicated in patients with congenital long QT syndrome, patients with a history of cardiac arrhythmias, patients taking other drugs that prolong the QT interval, and patients with known hypokalemia (i.e., low potassium levels) or hypomagnesemia (i.e., low magnesium levels) [155]. It is therefore of tremendous interest to develop safer alternatives to pimozide that minimize its hERG activity while retaining its therapeutic efficacy.

In this work, we apply the CardioGenAI framework to re-engineer pimozide for reduced hERG inhibition while preserving its pharmacological activity. The experimentally determined pIC_50_ value of pimozide for the hERG channel is 8.520, and the value that our regression model predicts is 7.629, a difference (0.891 pIC_50_) which is sufficiently small to be attributable to variance in experimental protocols used to obtain labels [156]. Our objective is to generate compounds with similar pharmacological properties, but with predicted hERG channel pIC_50_ values less than 6.0.

We therefore condition the molecular generation on the scaffold and physicochemical properties of pimozide, and filter out molecules with a predicted hERG channel pIC_50_ value greater than or equal to 6.0. This procedure is performed until 100 compounds are generated, which takes approximately one minute using an NVIDIA GeForce RTX 4050 GPU. We then compute descriptor vectors for pimozide and the filtered generated molecules, and then calculate the cosine similarity between the descriptor vector of pimozide and those of the generated molecules. In practice, many more molecules can be generated to create a molecular library for further screening.

**Fig. 4:**
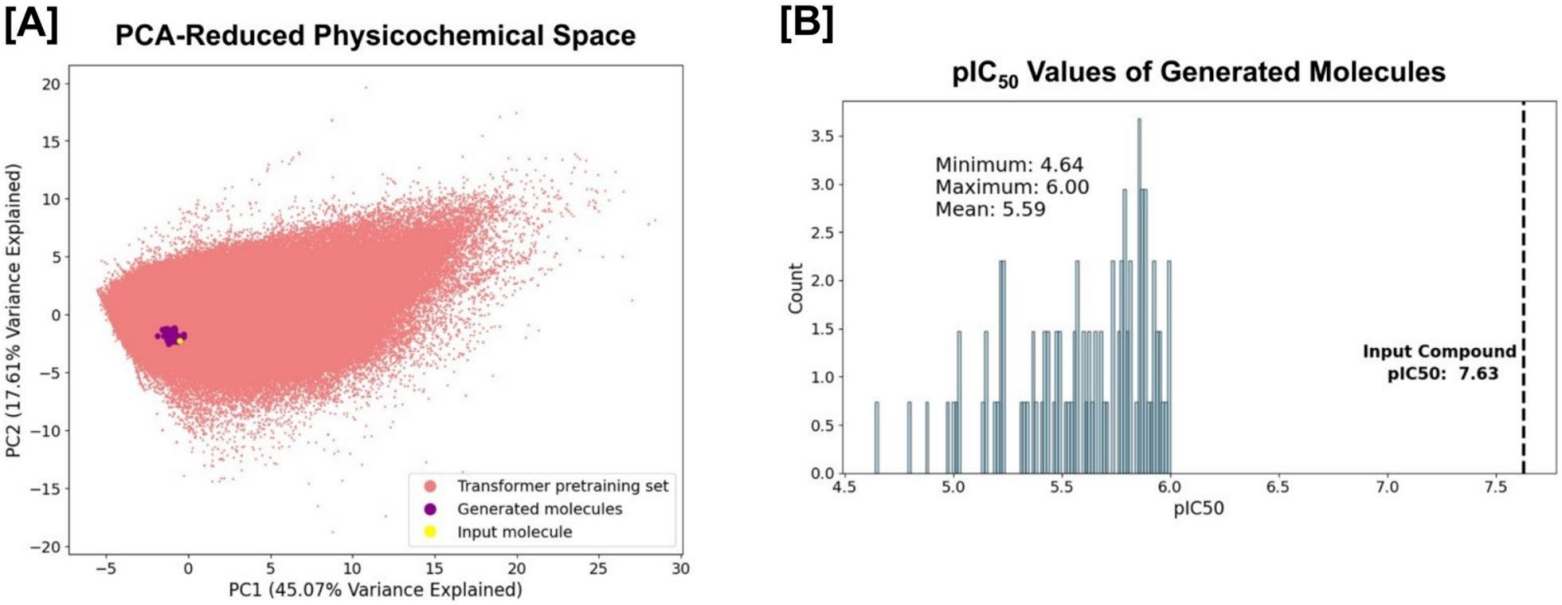
Visualization of the CardioGenAI framework applied to pimozide. The input molecule (pimozide), the 100 generated refined molecules, and the molecules in the training set for the transformer-based models (approximately 5 million datapoints), are projected into a principal component analysis (PCA)-reduced physicochemical-based space, shown in (**A**). Pimozide is colored yellow, the generated refined compounds are colored purple, and the compounds in the training set of the transformer-based models are colored red. The first two principal components explain 45.07% and 17.61% of the total variance, respectively. Clearly, the CardioGenAI framework is able to identify the region of physicochemical space corresponding to compounds that are similar to pimozide, yet exhibit significantly reduced activity against the hERG channel. The density of predicted pIC_50_ values against the hERG channel of the generated refined compounds as compared to that of pimozide is shown in (**B**). The distribution of generated compounds exhibits a maximum predicted pIC_50_ value of 6.00, with a mean of 5.59 and minimum of 4.64

We calculate the ten previously described physicochemical properties for pimozide, the 100 filtered generated molecules, and the molecules in the transformer training set, and then perform principal component analysis (PCA) to construct a lower-dimensional chemical space in which we can visually compare the filtered generated molecules to pimozide in relation to the broader transformer training set. Plotting the first two PCs reveals that the filtered generated molecules are closely aligned to pimozide, indicating that our framework successfully navigates the initially vast chemical space to propose compounds with similar physicochemical characteristics to pimozide but with reduced hERG activity (Fig. 4A; Figure S15 in Additional file 1). Additionally, the distribution of predicted pIC_50_ values of the generated compounds ranges from 4.64 to 6.00 with a mean value of 5.59, indicating significant reductions in hERG activity (Fig. 4B). The most similar generated molecules to pimozide are reported in Table S16 of Additional file 1.

**Fig. 5:**
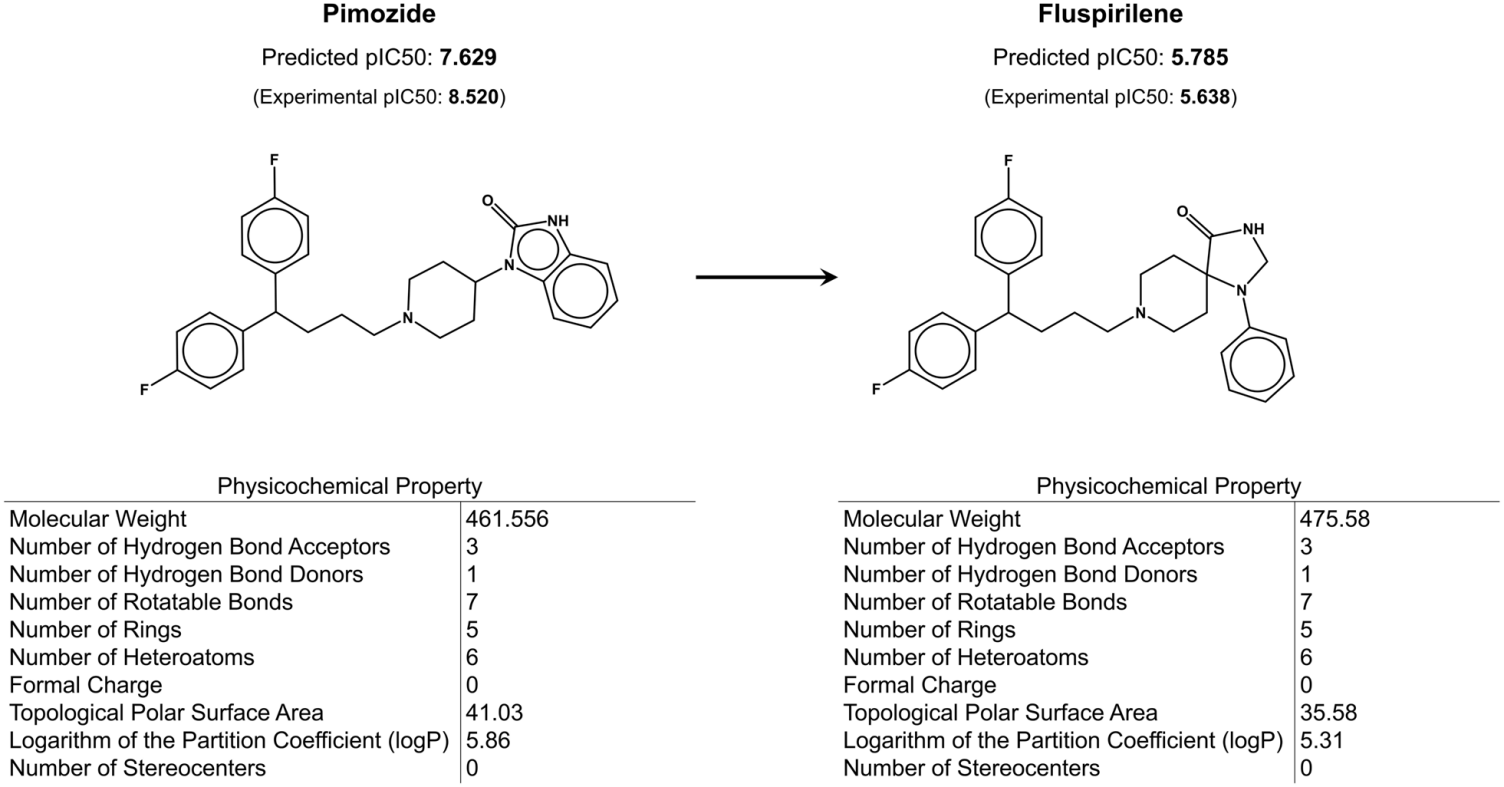
CardioGenAI framework applied to pimozide, an FDA-approved antipsychotic drug that has an experimental hERG pIC_50_ value of 8.520 (predicted: 7.629), and is reported to cause hERG channel blockade-induced QT interval prolongation and arrhythmias. CardioGenAI proposes 100 molecules, and among them is fluspirilene, a compound that belongs to the same class of drugs as pimozide but exhibits over 700-fold weaker binding to hERG (experimental pIC_50_ value is 5.638)

We analyze each of the 100 generated refined compound with respect to all of the compounds provided in the DrugCentral Postgres v14.5 database to identify any compounds approved by either the FDA, the European Medicines Agency (EMA), or the Pharmaceuticals and Medical Devices Agency of Japan (PMDA) [130, 131]. Remarkably, among the 100 filtered generated compounds is fluspirilene, a compound that belongs to the same class of drugs as pimozide (diphenylmethanes) and therefore presents a highly similar pharmacological profile [157]. Moreover, the experimental hERG pIC_50_ value of fluspirilene is 5.638 (predicted: 5.785), as compared to 8.520 (predicted: 7.629) for pimozide (Fig. 5), indicating a reduction in hERG activity by over 700-fold.

The reduced hERG activity of fluspirilene compared to pimozide can be attributed to the presence of an aromatic nitrogen-containing heterocyclic group in pimozide, which is absent in fluspirilene (Fig. 5). Aromaticity increases the basicity of the nitrogen, allowing for protonation and stronger electrostatic and π-cation interactions with the hERG channel. This aligns with prior literature and our observations (Sect. "Data Featurization") that basic, aromatic nitrogens are significant contributors to hERG activity [127].

This case study demonstrates the ability of the CardioGenAI framework to re-engineer a hERG-active compound for reduced hERG activity while preserving its pharmacological activity.

### Additional applications of the complete framework for hERG activity optimization

**Fig. 6:**
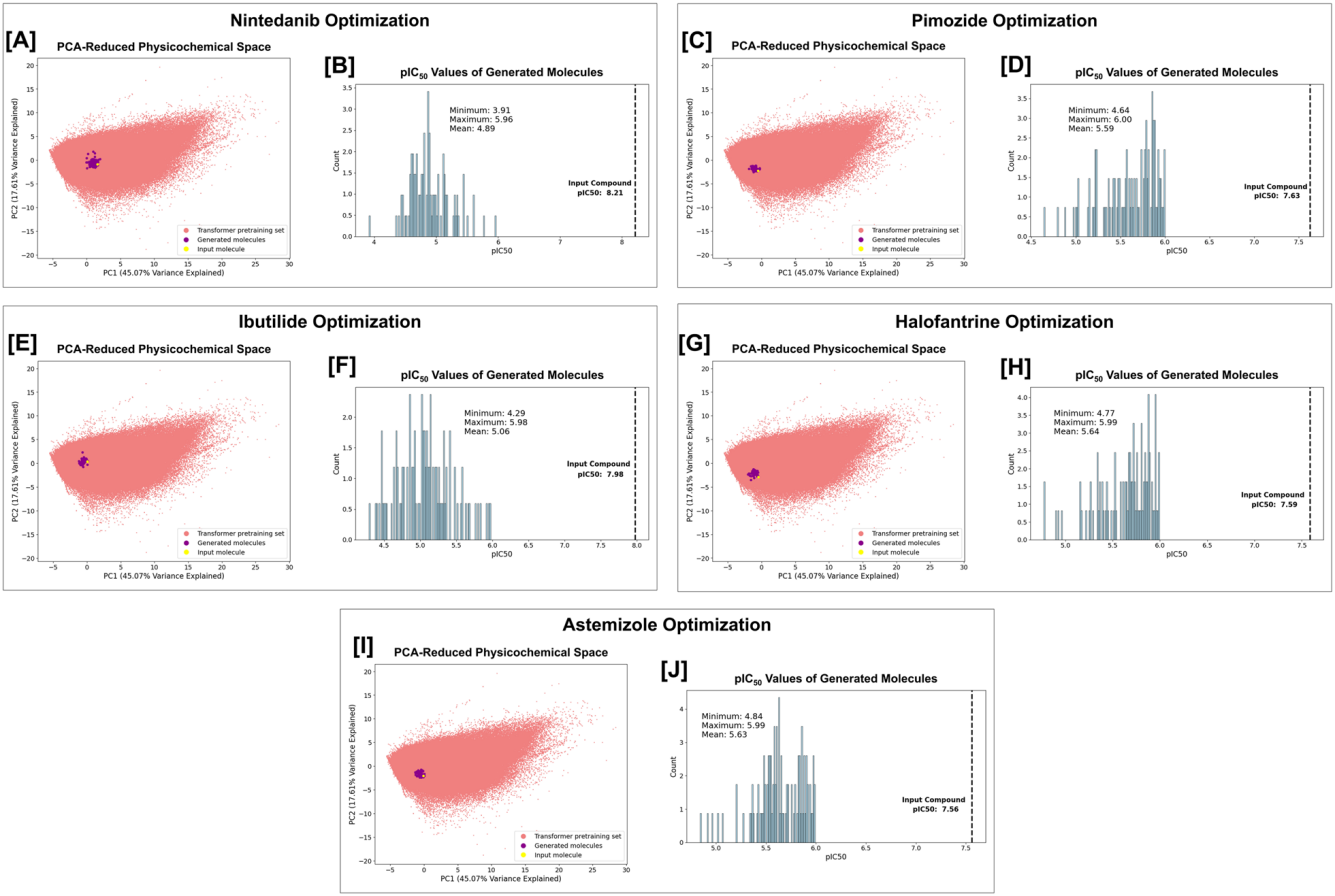
Visualization of the CardioGenAI framework applied to nintedanib (**A**, **B**), pimozide (**C**, **D**), ibutilide (**E**, **F**), halofantrine (**G**, **H**), and astemizole (**I**, **J**). In each application, the specified maximum predicted hERG pIC_50_ value of any of the generated compounds was set to 6.00. For each optimization, the input molecule, the 100 generated refined molecules, and the molecules in the training set for the transformer-based models (approximately 5 million datapoints), are projected into a principal component analysis (PCA)-reduced physicochemical-based space. The input compound is colored yellow, the generated refined compounds are colored purple, and the compounds in the training set of the transformer-based models are colored red. The first two principal components explain 45.07% and 17.61% of the total variance, respectively. In each case, the CardioGenAI framework is able to identify the region of physicochemical space corresponding to compounds that are similar to the input compound, yet exhibit significantly reduced activity against the hERG channel. The densities of predicted pIC_50_ values against the hERG channel of the generated refined compounds as compared to that of the respective input compound are shown in [B]. Relevant metrics are shown on each plot

In addition to re-engineering pimozide, we also apply the CardioGenAI framework to nintedanib, ibutilide, halofantrine, and astemizole. Collectively, including pimozide, these five compounds are those among the set of FDA-approved compounds provided by DrugCentral that have the highest predicted pIC_50_ values against the hERG channel. We show that for each drug, the framework is able to successfully generate compounds with similar physicochemical profiles and with significantly reduced activity against the hERG channel (Fig. 6).

### Applications of the complete framework for Na_V_1.5 and Ca_V_1.2 activity optimization

**Fig. 7:**
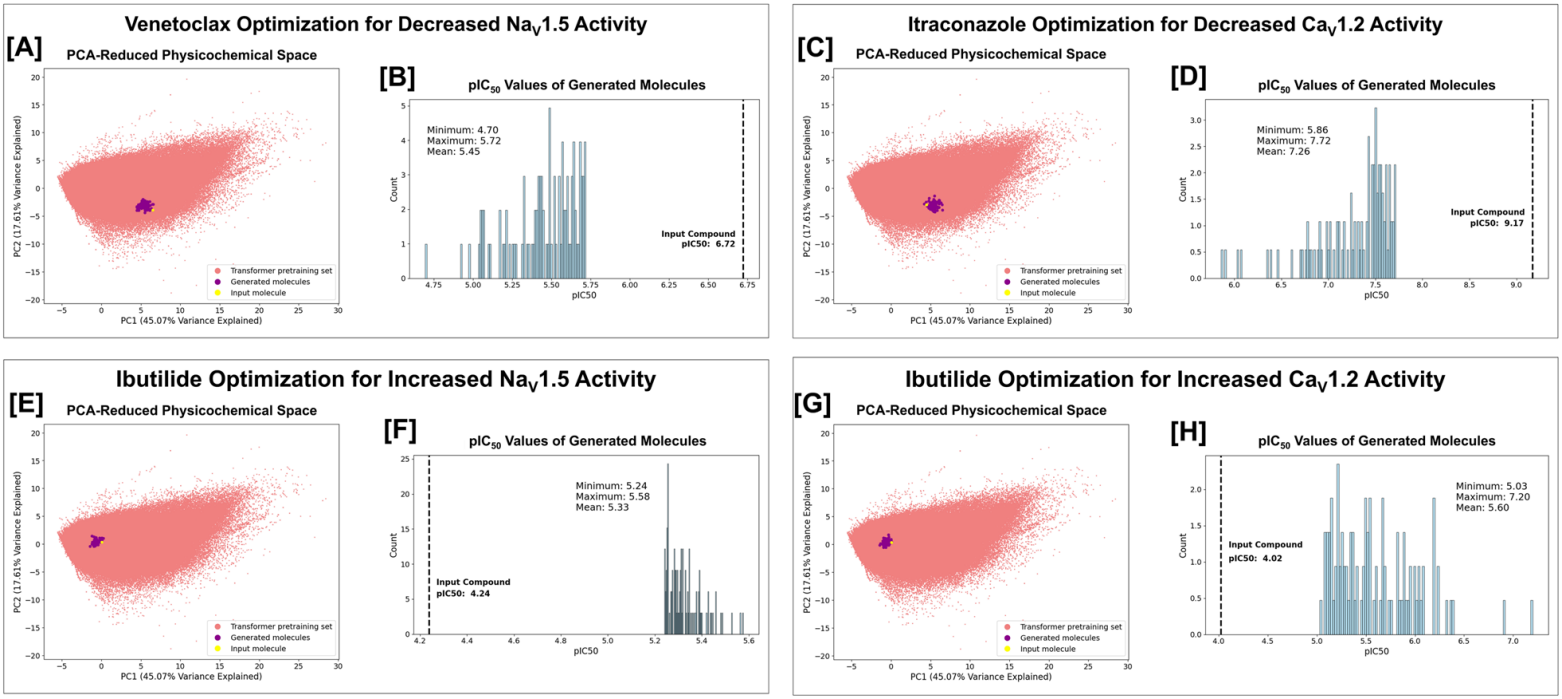
Visualization of the CardioGenAI framework applied to venetoclax (**A**, **B**), itraconazole (**C**, **D**), and ibutilide (**E**–**H**). In each case, the specified predicted cardiac ion channel pIC_50_ value for each of the generated compounds is set to be at least an improvement of one order of magnitude compared to that of the input compound. For each optimization, the input molecule, the 100 generated refined molecules, and the molecules in the training set for the transformer-based models (approximately 5 million datapoints), are projected into a principal component analysis (PCA)-reduced physicochemical-based space. The input compound is colored yellow, the generated refined compounds are colored purple, and the compounds in the training set of the transformer-based models are colored red. The first two principal components explain 45.07% and 17.61% of the total variance, respectively. For venetoclax, which has a predicted Na_V_1.5 pIC_50_ of 6.72, we reduce the Na_V_1.5 pIC_50_ by at least one order of magnitude for each generated compound (**B**). For itraconazole, which inhibits Ca_V_1.2 with a predicted pIC_50_ of 8.72, we reduce the Ca_V_1.2 pIC_50_ by at least one order of magnitude for each generated compound (**D**). For ibutilide, which has a predicted pIC_50_ for hERG, Na_V_1.5, and Ca_V_1.2 of 7.98, 4.24 and 4.02, respectively, we independently increase the Na_V_1.5 pIC_50_ by at least one order of magnitude for each generated compound (**F**) and increase the Ca_V_1.2 pIC_50_ by at least one order of magnitude for each generated compound (**H**). In each case, the CardioGenAI framework is able to identify the region of physicochemical space corresponding to compounds that are similar to the input compound, yet exhibit significantly improved activity against the respective cardiac ion channel. The densities of predicted pIC_50_ values of the generated refined compounds against the respective cardiac ion channel are shown. Relevant metrics are shown on each plot

Moreover, given that modulating Na_V_1.5 and Ca_V_1.2 channel activities may mitigate the arrhythmogenic potential induced by hERG channel blockade [6–8], and considering that activity against each of these two channels alone can present problems related to the cardiac action potential [10, 45], we demonstrate the ability of the framework to optimize compounds for enhanced Na_V_1.5 and Ca_V_1.2 profiles. Specifically, we assess the capabilities of the framework with respect to four independent objectives: (1) Increase the Na_V_1.5 activity of a compound that has high hERG activity but low Na_V_1.5 activity; (2) Increase the Ca_V_1.2 activity of a compound that has high hERG activity but low Ca_V_1.2 activity; (3) Decrease the Na_V_1.5 activity of a compound that has high Na_V_1.5 activity; (4) Decrease the Ca_V_1.2 activity of a compound that has high Ca_V_1.2 activity. For cases (1) and (2), we chose to re-engineer ibutilide, which has a predicted pIC_50_ for hERG, Na_V_1.5, and Ca_V_1.2 of 7.98, 4.24 and 4.02, respectively. For case (3), we chose venetoclax, which has a predicted Na_V_1.5 pIC_50_ of 6.72. For case (4), we chose itraconazole, which inhibits Ca_V_1.2 with a predicted pIC_50_ of 9.17. The CardioGenAI framework is able to successfully improve the cardiac ion channel activity by at least one order of magnitude in each case for every generated refined compound while ensuring that the generated compounds are physicochemically similar to the respective input drug. The results for each of these four cases are presented in Fig. 7.

## Customizing the CardioGenAI framework for company-specific industrial applications

Pharmaceutical companies have begun to leverage generative AI-based methods for specific tasks within the earlier stages of drug discovery pipelines [158]. In order to facilitate integration of CardioGenAI into drug discovery workflows, all of the software is entirely open-source and the framework is designed to be easily customizable. Companies can therefore incorporate desired functionality, and retrain all of the models on their internal data. It is expected that large pharmaceutical companies will significantly benefit from retraining the models, given that their internal data is likely more comprehensive and subject to significantly less experimental variance than the publicly available datasets used to initially train the models.

With respect to the incorporation of additional functionality into the framework, CardioGenAI is designed such that predictive models can easily be integrated into the filtering phase along with the cardiac ion channel activity prediction models. For instance, a team of medicinal chemists will likely adhere to synthesis-related criteria; a rule-based filter, or a model fit to these criteria, can easily be incorporated. The objective of such a model could be to identify compounds that can be produced given an initial compound and feasible synthetic pathways, or to predict a synthetic accessibility score for a given compound. In theory, any predictive model can be integrated into the framework (e.g., for predicting on-target activity, solubility, metabolic stability, bioavailability, etc.).

Because synthesizability is arguably the most important characteristic of a proposed compound, additional steps can be taken, aside from incorporating more models, to ensure that the proposed compounds are in accordance with a company’s specific synthesis capabilities. For instance, the dataset used to train the generative autoregressive transformer could be curated to contain only compounds that a company deems sufficiently synthesizable, thereby biasing the generative component of the framework to only propose compounds that are akin to those that satisfy these synthesizability standards. Additionally, rather than defining the chemical space based on RDKit descriptors to identify molecules that are physicochemically similar to the input molecule, the space can be designed such that nearby molecules are easily synthesizable.

In the current implementation, RDKit is used to validate the proposed molecules generated by the framework, ensuring that molecular representations conform to basic valence and bonding rules. However, it does not assess chemical plausibility beyond these criteria. As such, some structures may be valid according to RDKit but exhibit features that are chemically improbable. To address this, the framework can easily be augmented with additional criteria applied at the generation stage to enforce properties such as thermodynamic stability or broader chemical plausibility. These enhancements allow users to refine the generative process further, ensuring that proposed compounds align with expectations.

## Summary

Although numerous generative models have demonstrated the ability to produce molecules with prespecified drug-like properties, as well as molecules with desired on-target potency, there has been comparatively less effort devoted to developing and applying generative models for off-target potency optimization. In this work, we present an ML-based framework for re-engineering hERG-active compounds for reduced hERG activity while preserving their pharmacological activity. The method utilizes an autoregressive transformer-based generative model to produce molecules conditioned on the molecular scaffold and set of physicochemical properties of the input molecule. The generated ensemble is filtered based on hERG, Na_V_1.5 and Ca_V_1.2 activity using state-of-the-art discriminative deep learning models. A physicochemical-based space is then constructed from the filtered generated distribution and the input molecule, where nearby molecules have similar physicochemical profiles, thus facilitating the identification of molecules with similar pharmacological activity to the input molecule but with reduced hERG liability. We applied the framework to pimozide, an FDA-approved antipsychotic agent that demonstrates high affinity to the hERG channel, and generated a compound of the same class of drugs that has a significantly lower hERG pIC_50_ value as indicated by both predicted and experimental values. Furthermore, we demonstrated the framework's ability to optimize hERG, Na_V_1.5 and Ca_V_1.2 profiles of multiple FDA-approved compounds while maintaining the physicochemical nature of the original drugs. In addition, the state-of-the-art performances of the hERG, Na_V_1.5, and Ca_V_1.2 activity prediction models support their independent utility as effective components of virtual screening campaigns.

## Technical implementation details

The transformer-based models and the feed-forward networks in the discriminative models were built using PyTorch [159]. The parameters of the transformer-based models were optimized using the Sophia optimizer [151]. The GAT components of the discriminative models were built using PyTorch Geometric [160]. The hyperparameters of the discriminative models were optimized using Optuna [161]. The hyperparameters that were optimized include: batch size, learning rate, weight decay, the number of GAT attention heads used in the graph model, the output dimension of the GAT mechanism used in the graph model, and the dropout rate applied to the fully connected components of the complete architecture. SMILES canonicalization, as well as the calculations of physicochemical properties and molecular scaffolds were performed using RDKit [113]. Scikit-learn was used to calculate pairwise mutual information between chemical features and cosine similarity between descriptor vectors, as well as to perform PCA [162].

## Supplementary Information

Supplementary Material 1. Details regarding the datasets used, model trainings, additional analyses of the models, and the refined drug candidates.

## Data Availability

All of our software is available as open-source at https://github.com/gregory-kyro/CardioGenAI. Users can easily run the complete CardioGenAI framework, perform inference with the discriminative models, and reproduce the figures in this manuscript. Additionally, we provide all of the data we use, as well as the parameters for each of our trained models.

## Abbreviations

hERG: Human Ether-à-go-go-Related Gene
TdP: Torsade de Pointes
CiPA: The Comprehensive In Vitro Proarrhythmia Assay
FDA: U.S. Food and Drug Administration
Na_V_1.5: Voltage-gated sodium ion channel subtype 1.5
Ca_V_1.2: Voltage-gated calcium ion channel subtype 1.2
ML: Machine learning
AI: Artificial intelligence
LogP: Logarithm of the partition coefficient between n-octanol and water
TPSA: Topological polar surface area
ECFP4: Extended-Connectivity Fingerprint with a diameter of 4 bonds
GAT: Graph attention network
AC: Accuracy
SN: Sensitivity
SP: Specificity
CCR: Correct classification rate
MCC: Matthew’s correlation coefficient
AUC: Area under the curve
*Q*: Query vector
*K*: Key vector
*V*: Value vector
CNS: Central nervous system
PCA: Principal component analysis
