# Supplementary material for: CardioGenAI: a machine learning-based framework for re-engineering drugs for reduced hERG liability
Source: J Cheminform. 2025 Mar 5;17:30. doi: 10.1186/s13321-025-00976-8 (PMC11881490; doi:10.1186/s13321-025-00976-8)
Supplement: Supplementary file 1 — Supplementary Material 1. Details regarding the datasets used, model trainings, additional analyses of the models, and the refined drug candidates. [file 13321_2025_976_MOESM1_ESM.pdf]

Supplementary Information

CardioGenAI: A Machine Learning-Based Framework for Re-Engineering Drugs for Reduced hERG Liability

Gregory W. Kyro[1,2,*], Matthew T. Martin[2], Eric D. Watt[2], Victor S. Batista[1,*]

[1] Department of Chemistry, Yale University, New Haven, Connecticut 06511

[2] Drug Safety Research & Development, Pfizer Research & Development, Groton, Connecticut 06340

[*] Indicates corresponding author

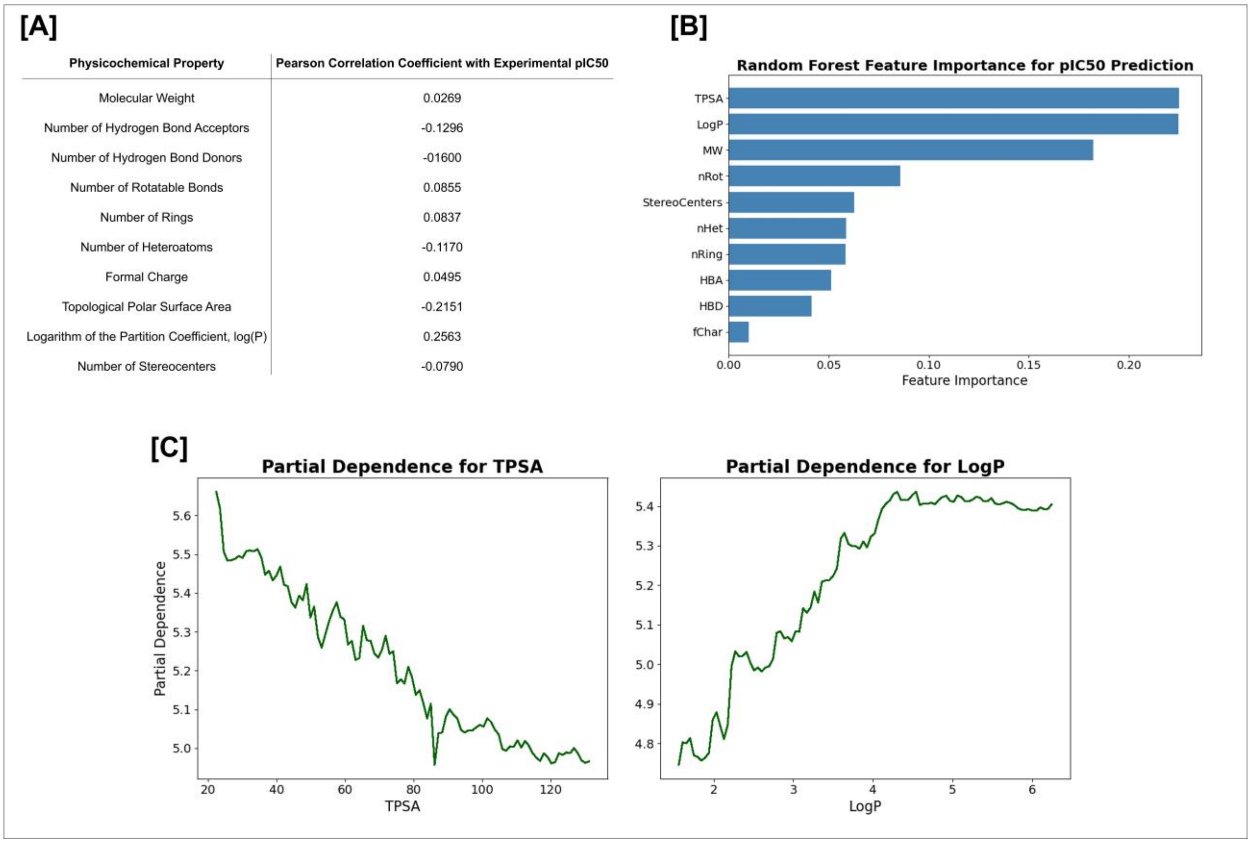

[A]

Physicochemical Property	Pearson Correlation Coefficient with Experimental pIC50
Molecular Weight	0.0269
Number of Hydrogen Bond Acceptors	-0.1296
Number of Hydrogen Bond Donors	-01600
Number of Rotatable Bonds	0.0855
Number of Rings	0.0837
Number of Heteroatoms	-0.1170
Formal Charge	0.0495
Topological Polar Surface Area	-0.2151
Logarithm of the Partition Coefficient, log(P)	0.2563
Number of Stereocenters	-0.0790

Figure S1. Analysis of the relationships between physicochemical properties and experimental hERG channel pIC_{50} values. In [A], Pearson correlation is shown between each physicochemical property and experimental pIC_{50} values obtained from the training set used. There are noteworthy correlations with pIC_{50} for topological polar surface area (TPSA) and LogP. A random forest model with 100 estimators was fit to the data to predict pIC_{50} values, and the importance of each feature was then deduced. In [B], the feature importance of each physicochemical property is shown. In [C], the partial dependences for TPSA ($Å^2$) and LogP are shown. For LogP, there is an initial positive trend were an increase in LogP corresponds to an increase in PIC_{50} values up to a LogP value of approximately 4. For TPSA, as TPSA increases, pIC_{50} values generally decrease.

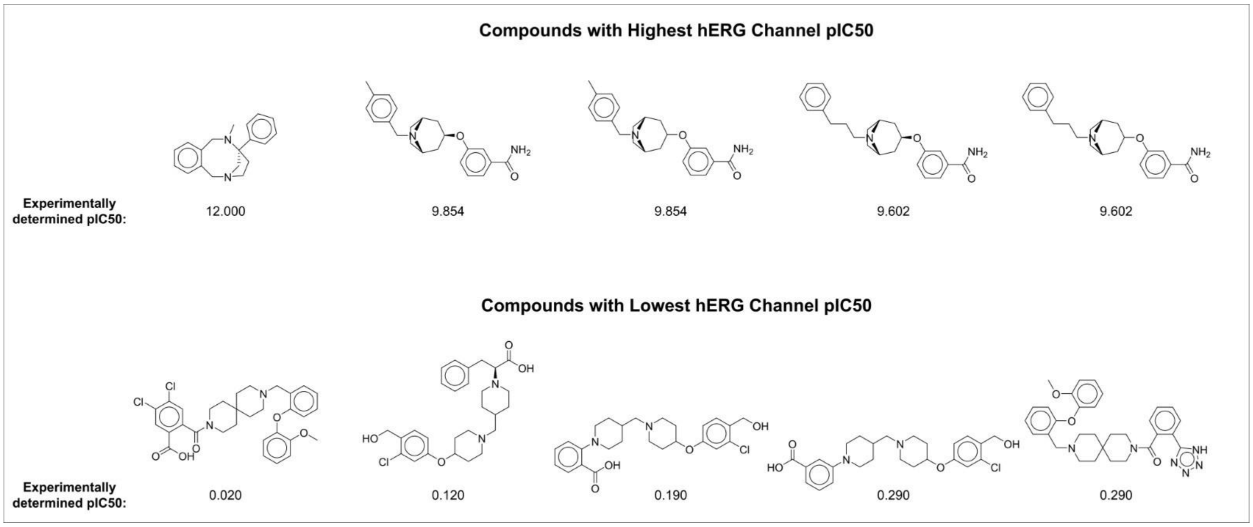

Figure S2. Display of the five compounds with highest hERG channel activity, as well as the five compounds with the lowest hERG channel activity of compounds in the hERG channel training set used. Each molecule is labeled with the corresponding experimentally determined hERG channel pIC_{50}. Molecules are rendered with ChemDraw v23.1.1.

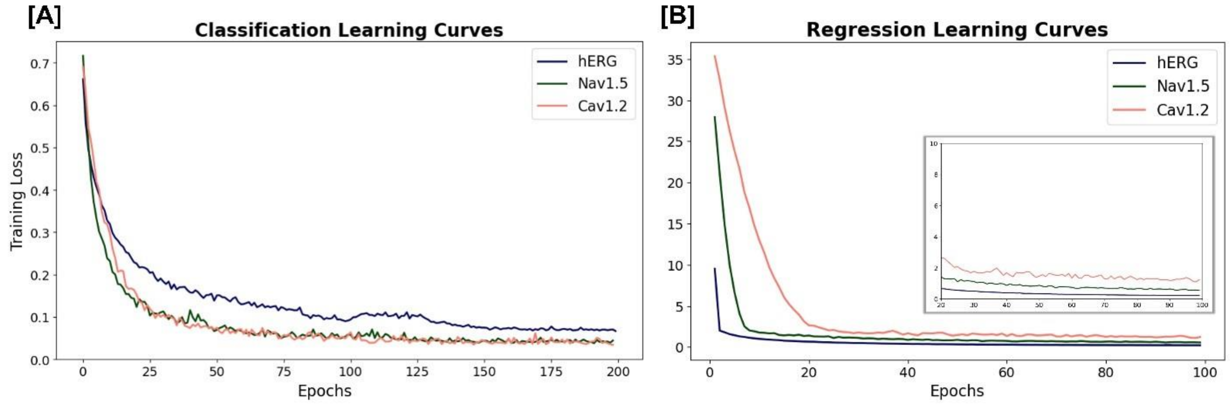

Figure S3. Learning curves for hERG, Nav1.5 and Cav1.2 cardiac ion channel [A] classification and [B] regression models. The classification models were trained with binary cross entropy loss and the AdamW optimizer for 200 epochs. Regression models were trained analogously but using mean squared error loss for 100 epochs.

Table S4. Performance regarding each possible feature-representation combination for binary classification of hERG channel blockers.

Feature Representations	AC	SN	SP	F1	CCR	MCC
Transformer feature vector + Graph + Fingerprint	**83.5**	**86.2**	**80.3**	**85.1**	**83.2**	**66.7**
Transformer feature vector + Fingerprint	80.4	82.9	77.3	82.3	80.1	60.4
Transformer feature vector + Graph	80.0	82.9	76.4	81.9	79.6	59.5
Graph + Fingerprint	78.6	80.9	75.9	80.6	78.4	56.8
Transformer feature vector	77.7	83.3	70.9	80.4	77.1	54.9
Fingerprint	76.6	79.3	73.4	78.8	76.3	52.7
Graph	74.4	87.8	58.1	79.0	73.0	48.6

[a] The evaluation set used is that developed by Arab et al.; compounds in the evaluation set have a structural similarity (as determined by pairwise Tanimoto similarity between 2048-bit Morgan fingerprints) no greater than 0.70 to any compound in the corresponding training or validation sets.
[b] The top value achieved for each metric is shown in bold.
[c] Accuracy (AC), sensitivity (SN), specificity (SP), F1-score (F1), correct classification rate (CCR), and Matthew's correlation coefficient (MCC) are shown.

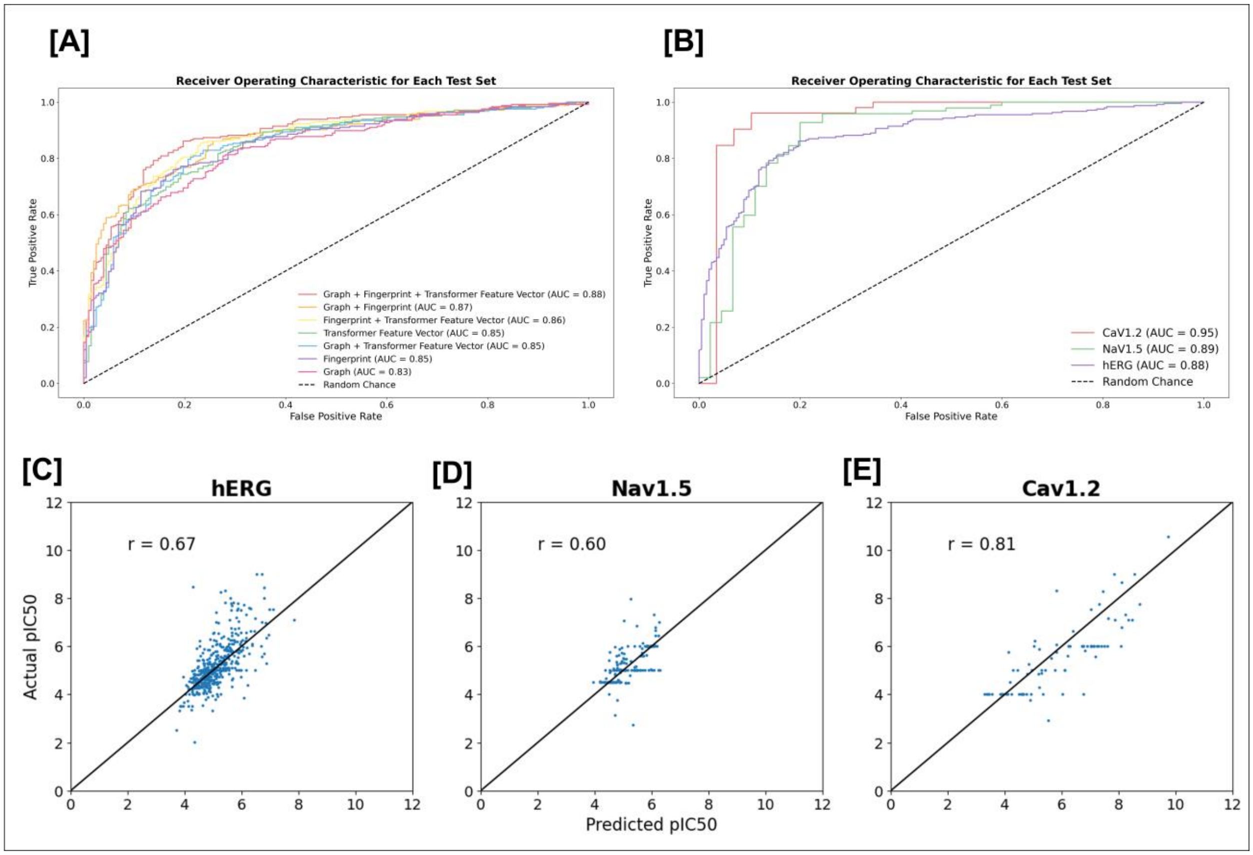

Figure S5. Evaluation of classification and regression models for cardiac ion channel blocker prediction. [A] Receiver operating characteristic (ROC) curve for evaluation on the hERG benchmark presented by Arab et al. Results regarding each feature-representation combination are shown with the corresponding area under the curve (AUC). [B] ROC curve for evaluation on the hERG, $Na_V1.5$ and $Ca_V1.2$ channel benchmarks. Scatter plots depicting actual pIC_{50} as a function of predicted pIC_{50}, with the corresponding Pearson correlation coefficient (r), shown for evaluation on the [C] hERG, [D] $Na_V1.5$, and [E] $Ca_V1.2$ channel benchmark sets.

Table S6. Performance of the hERG, $Na_V1.5$ and $Ca_V1.2$ regression models on the benchmarks presented by Arab et al.

Channel	R^2	Q^2_{F1}	Q^2_{F2}	Q^2_{F3}	CCC	r^2_m	RMSE	MAE
hERG	0.439	0.439	0.439	-20.922	0.606	0.116	0.741	0.509
$Na_V1.5$	0.323	0.327	0.323	-6.060	0.576	0.060	0.620	0.429
$Ca_V1.2$	0.536	0.541	0.536	-2.597	0.784	0181	1.006	0.821

[a] The evaluation sets used are those developed by Arab et al.; compounds in the evaluation set have a structural similarity (as determined by pairwise Tanimoto similarity between 2048-bit Morgan fingerprints) no greater than 0.70 to any compound in the corresponding training or validation sets.
[b] The coefficient of determination (R^2), predictive squared correlation coefficient for the first fold (Q^2_{F1}), predictive squared correlation coefficient for the second fold (Q^2_{F2}), predictive squared correlation coefficient for the third fold (Q^2_{F3}), concordance correlation coefficient (CCC), squared correlation coefficient for model (r^2_m), root mean square error (RMSE), and mean absolute error (MAE) are shown.

Table S7. Pearson correlation between physicochemical properties and predicted pIC_{50} values of compounds in each respective test set.

Physicochemical Property	hERG	$Na_V1.5$	$Ca_V1.2$
Molecular Weight	0.146	0.118	**0.444**
Number of Hydrogen Bond Acceptors	-0.159	-0.239	**0.621**
Number of Hydrogen Bond Donors	-0.114	**-0.593**	-0.002
Number of Rotatable Bonds	**0.327**	-0.294	**0.318**
Number of Rings	0.080	0.219	**-0.315**
Number of Heteroatoms	-0.081	0.095	**0.555**
Formal Charge	0.192	NaN	0.234
Topological Polar Surface Area, TPSA	-0.273	**-0.545**	**0.581**
Logarithm of the Partition Coefficient, log(P)	**0.321**	**0.406**	0.233
Number of Stereocenters	-0.169	-0.085	-0.182

[a] Compounds in the evaluation set have a structural similarity, as determined by pairwise Tanimoto similarity between 2048-bit Morgan fingerprints, no greater than 0.70 to any compound in the corresponding training or validation sets.

[b] NaN indicates that all values in the set are constant. In the case of Formal Charge for the $Na_V1.5$ channel, all compounds in the test set have a value of 0.

[c] Pearson correlation coefficient values with a magnitude greater than 3.00 are shown in bold.

Table S8. Y-randomization test applied to each of the classification models.

**Channel**	**AC**	**SN**	**SP**	**F1**	**CCR**	**MCC**
hERG	55.0	100.0	0.5	70.9	50.2	5.2
$Na_V1.5$	68.3	100.0	0.0	81.2	50.0	0.0
$Ca_V1.2$	65.4	100.0	3.4	78.8	51.7	15.0

[a] Compounds in the evaluation set have a structural similarity, as determined by pairwise Tanimoto similarity between 2048-bit Morgan fingerprints, no greater than 0.70 to any compound in the corresponding training or validation sets.

[b] Accuracy (AC), sensitivity (SN), specificity (SP), F1-score (F1), correct classification rate (CCR), and Matthew's correlation coefficient (MCC) are shown.

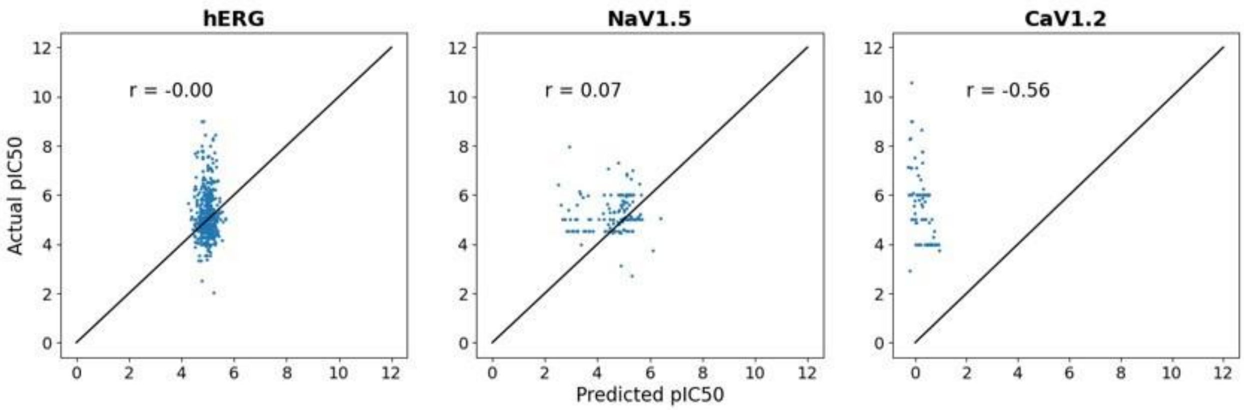

Figure S9. Y-randomization test applied to each of the regression models. Scatter plots depicting actual pIC_{50} as a function of predicted pIC_{50}, with the corresponding Pearson correlation coefficient (r), shown for evaluation on the hERG, $Na_V1.5$, and $Ca_V1.2$ channel benchmark sets.

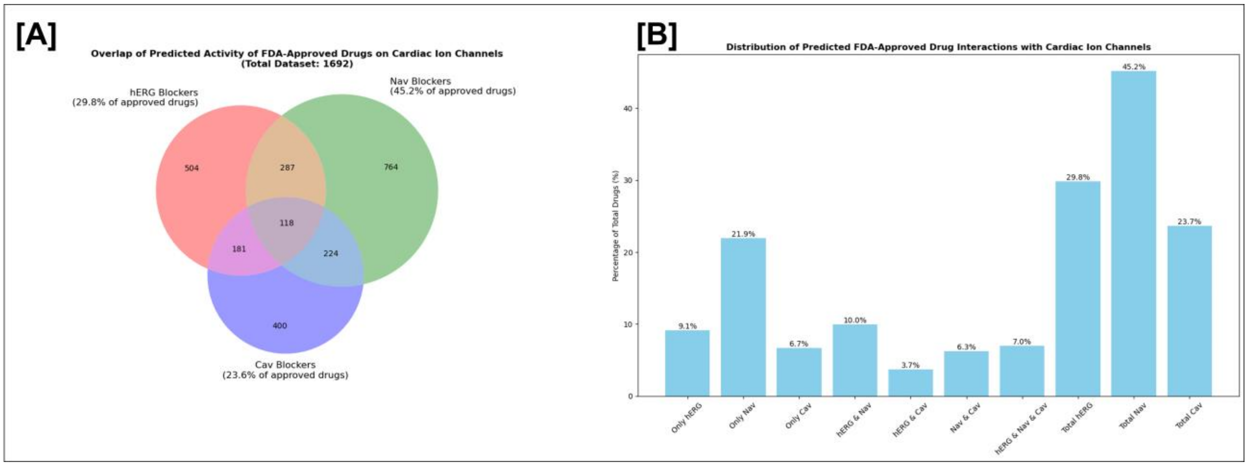

Figure S10. Application of cardiac ion channel classification models to a subset of FDA-approved drugs obtained from DrugCentral. A Venn diagram [A] and bar plot [B] are shown for the screening results of the 1692 total compounds.

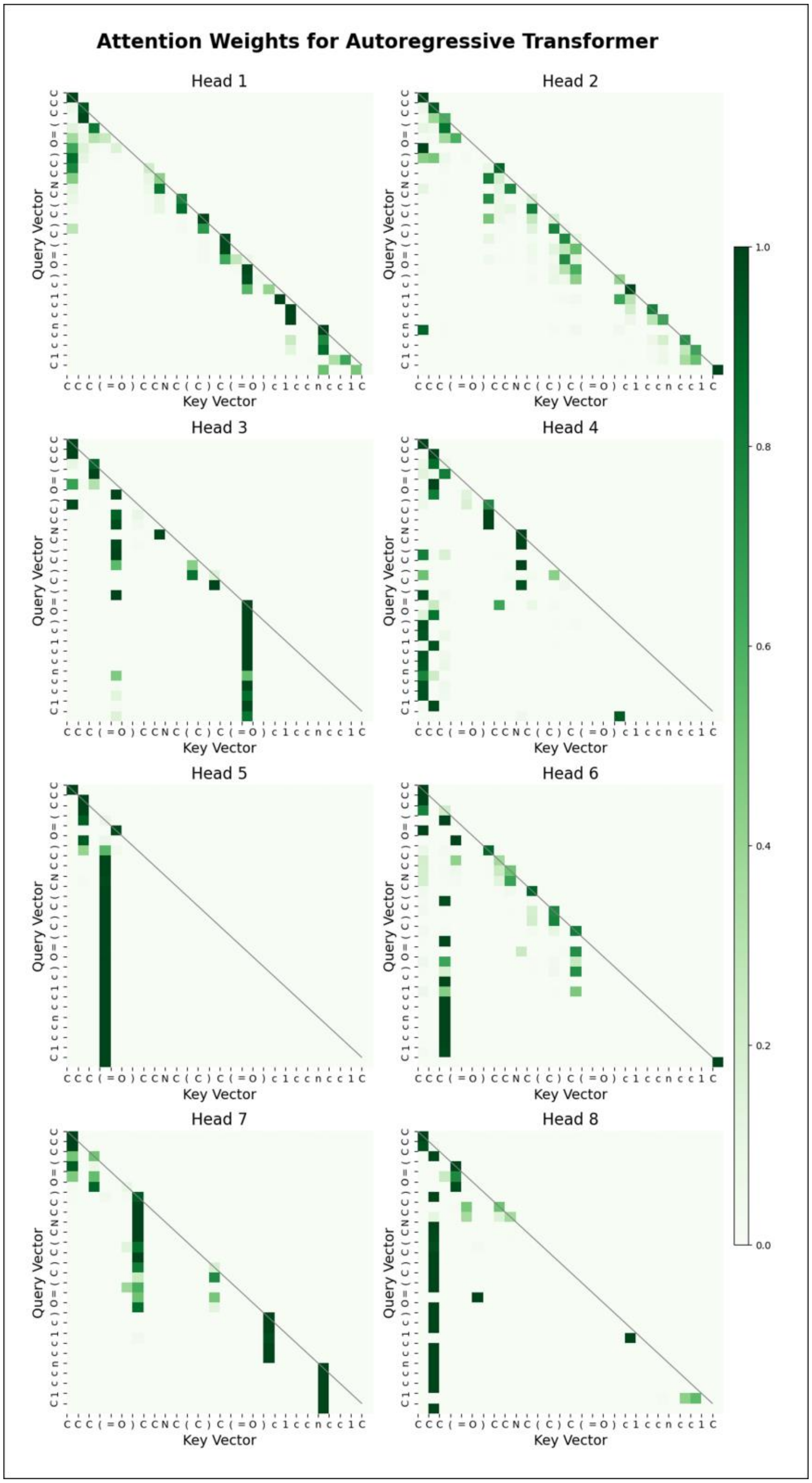

Figure S11. Representative attention maps depicting the distribution of attention weights extracted from the autoregressive transformer trained on SMILES strings, with each subplot corresponding to one of the eight heads in the model's attention layer. Weights are shown for the SMILES string: "CCC(=O)CCNC(C)C(=O)c1ccncc1C".

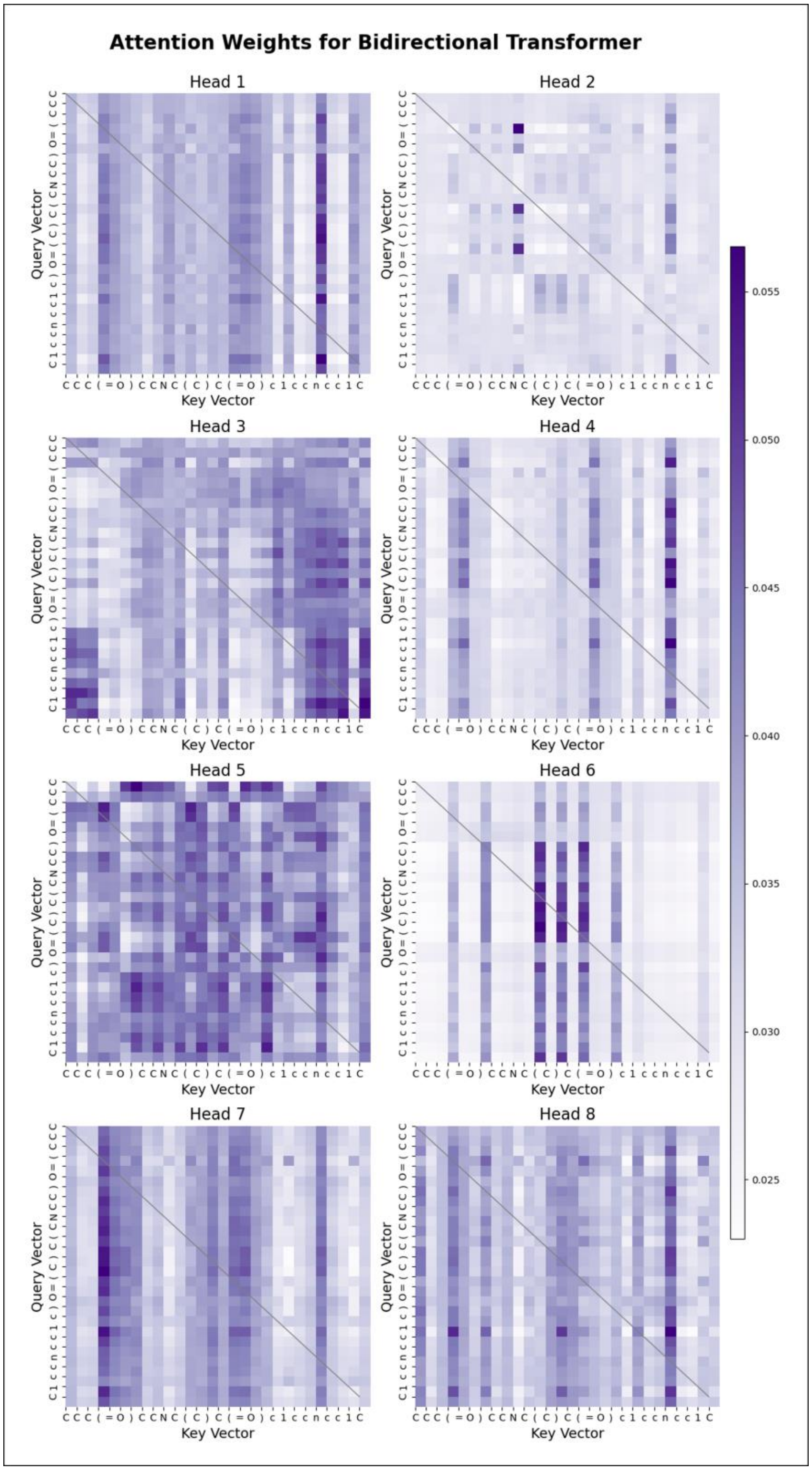

Figure S12. Representative attention maps depicting the distribution of attention weights extracted from the bidirectional transformer trained on SMILES strings, with each subplot corresponding to one of the eight heads in the model's attention layer. Weights are shown for the SMILES string: "CCC(=O)CCNC(C)C(=O)c1ccncc1C".

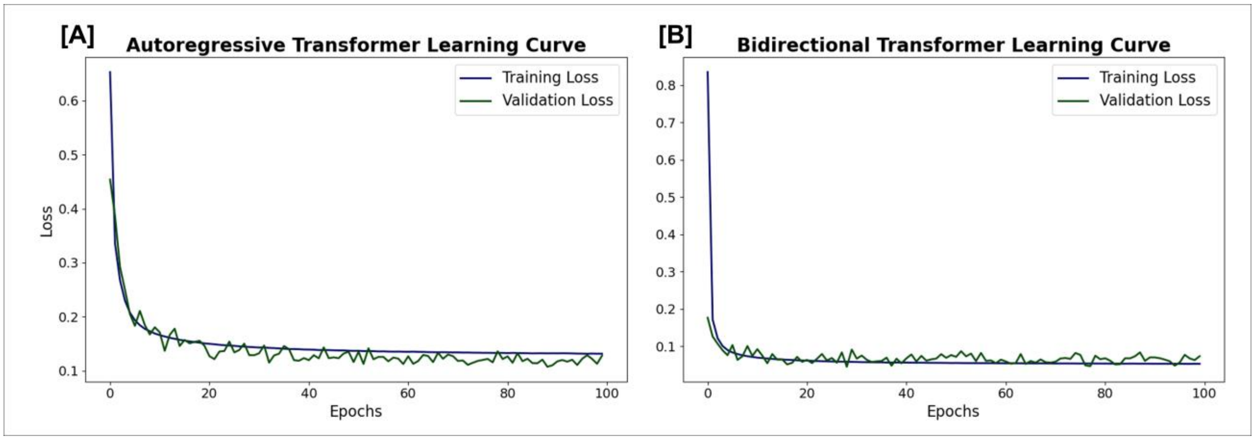

Figure S13. Learning curves for the [A] autoregressive transformer and [B] bidirectional transformer models. The autoregressive transformer is trained for next-token prediction, and the bidirectional transformer is trained for masked-token prediction. Both models are trained with cross entropy loss and the Sophia optimizer for 100 epochs.

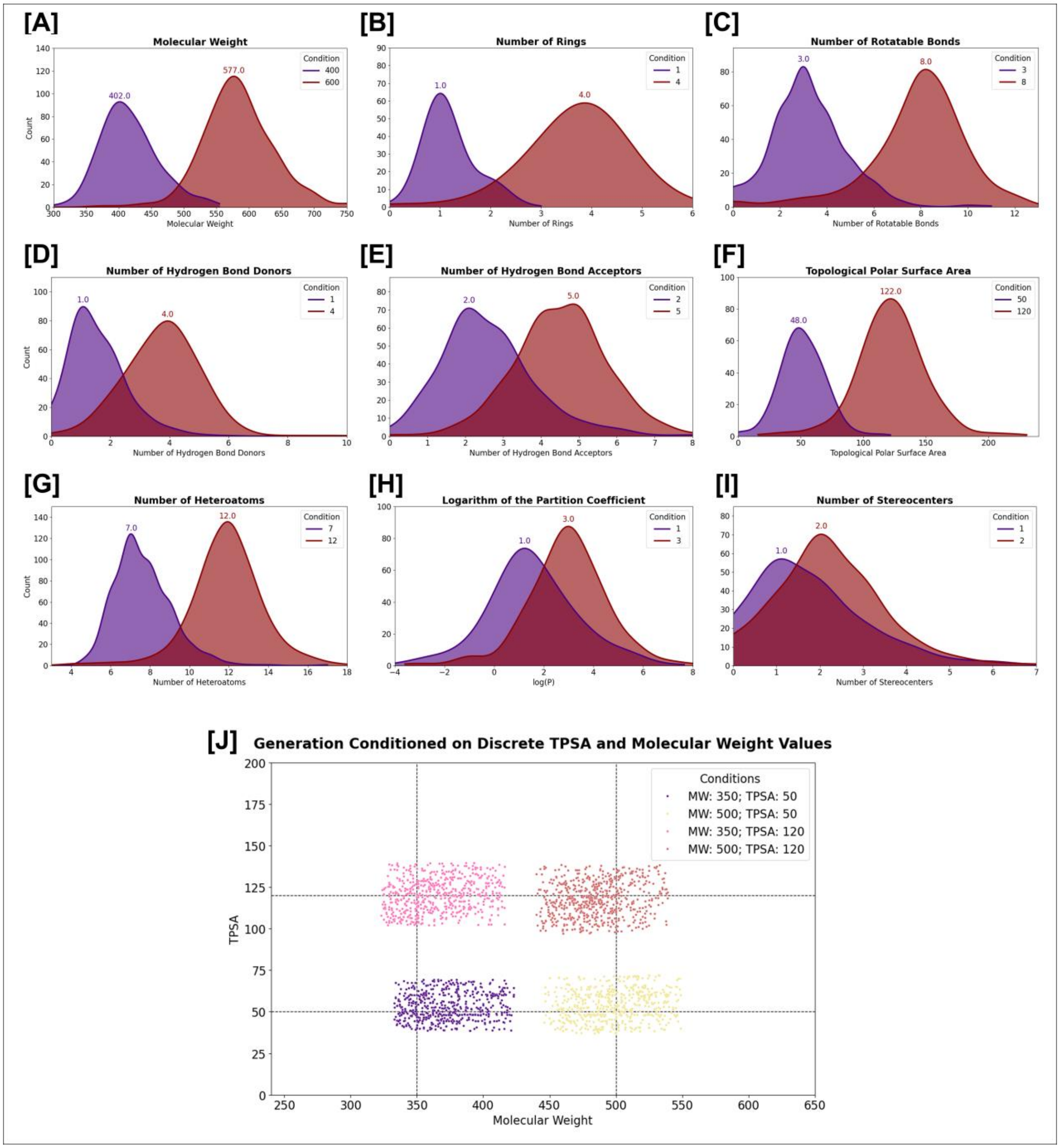

Figure S14. Probing the ability of the generative model to generate distributions of molecules with desired conditions. Results are shown for generations conditioned on different discrete values for [A] molecular weight ($\frac{g}{mol}$), [B] number of rings, [C] number of rotatable bonds, [D] number of hydrogen bond donors, [E] number of hydrogen bond acceptors, [F] topological polar surface area ($Å^2$), [G] number of heteroatoms, [H] LogP (logarithm of the partition coefficient), and [I] number of stereocenters. In [J], the generations are conditioned based on different value combinations of topological polar surface area (TPSA) and molecular weight. For each of the four pairs of conditions, values above/below two standard deviations greater/less than the mean value of each metric are excluded to emphasize the locations of the distribution means.

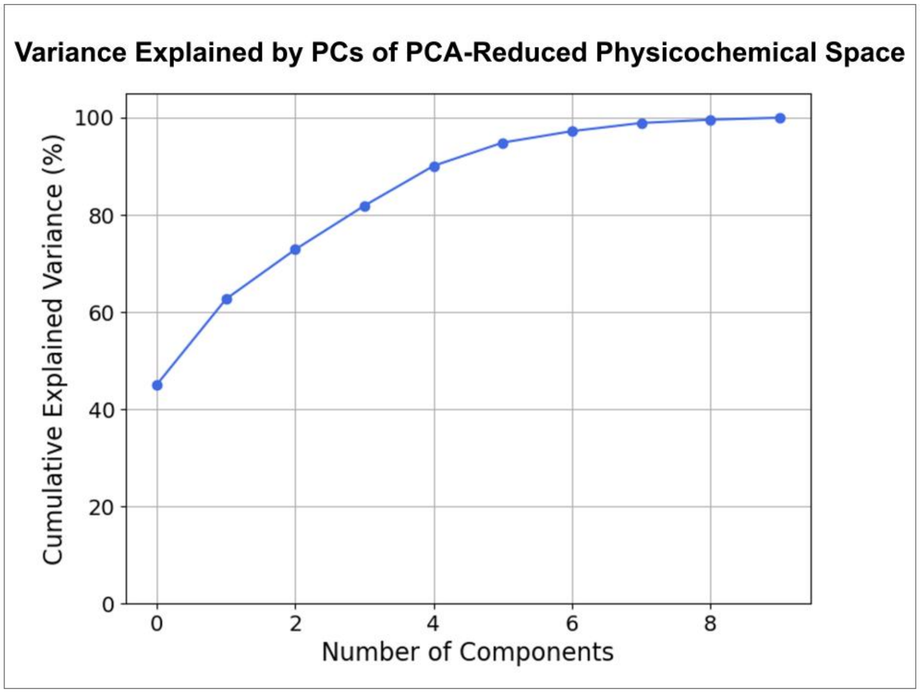

Figure S15. Cumulative variance as a function of principal component for principal component analysis (PCA) of physicochemical-based chemical space. For the input cardiotoxic molecule (pimozide), the generated molecules (100 datapoints), and the molecules in the pretraining set for the autoregressive transformer-based generative model (approximately 5 million datapoints), physicochemical properties are calculated, and PCA is performed to generate a lower-dimensional representation of chemical space.

Table S16. CardioGenAI methodology applied to pimozide, an FDA-approved antipsychotic drug that has a predicted hERG-channel pIC_{50} of 7.629, and is reported to cause hERG channel blockade-induced arrhythmias (Table 4). 100 molecules are generated, and among them is fluspirilene, a compound that belongs to the same class of drugs as pimozide and therefore has a similar primary therapeutic mode of action, but exhibits significantly less hERG-channel activity (5.785 pIC_{50}). Included in the table are the five most similar generated compounds to pimozide in terms of cosine similarity between molecular descriptor vectors.

Similarity Rank	SMILES String	Cosine Similarity	Predicted pIC_{50}
Input Molecule	O=c1[nH]c2ccccc2n1C1CCN(CCCC(c2ccc(F)cc2)c2ccc(F)cc2)CC1 (pimozide)	1.000	7.629
1	Fc1ccc(CCCN2CCc3c[nH]c(n3)C2)c(N2CCC(c3ccccc3)CC2)c1	0.980	5.367
2	O=C(Nc1ccccc1)N(CCCN1CCCC1)CC1(c2ccc(F)cc2)CCC1	0.977	5.904
3	O=C(NCc1ccc(F)cc1)N(CCCN1CCCC1)c1cccc2ccccc12	0.976	5.794
4	O=C1NCN(c2ccccc2)C12CCN(CCCC(c1ccc(F)cc1)c1ccc(F)cc1)CC2 (fluspirilene)	0.948	5.785
5	Fc1cccc(Cc2ncc(C3(N4CCC(Cc5ccccc5)CC4)CCOCC3)[nH]2)c1	0.946	5.201

Running the CardioGenAI Framework

To optimize a cardiotoxic compound with CardioGenAI, utilize the `optimize_cardiotoxic_drug` function from the `Optimization_Framework` module:

```
from src.Optimization_Framework import optimize_cardiotoxic_drug

optimize_cardiotoxic_drug(input_smiles,
                          herg_activity,
                          nav_activity,
                          cav_activity,
                          n_generations,
                          device)
```

- `input_smiles (str)`: The input SMILES string of the compound that you seek to optimize for reduced cardiac ion channel activity.
- `herg_activity (tuple or str)`: hERG activity for which to filter. If the entry is a string, it must be either 'blockers' or 'non-blockers'. If it is a tuple, it must indicate a range of activity values.
- `nav_activity (tuple or str)`: NaV1.5 activity for which to filter. If the entry is a string, it must be either 'blockers' or 'non-blockers'. If it is a tuple, it must indicate a range of activity values.
- `cav_activity (tuple or str)`: CaV1.2 activity for which to filter. If the entry is a string, it must be either 'blockers' or 'non-blockers'. If it is a tuple, it must indicate a range of activity values.
- `n_generations (int)`: The number of optimized drug candidates to generate. Default is 100.
- `device (str)`: The device to use for the optimization. Must be either 'gpu' or 'cpu'. Default is 'gpu'.

Figure S17. Python function to run the complete CardioGenAI framework.

Performing Inference with the Discriminative Models

To predict activity against the hERG, NaV1.5 and CaV1.2 channels, utilize the `predict_cardiac_ion_channel_activity` function from the `Discriminator` module:

```
from src.Discriminator import predict_cardiac_ion_channel_activity

predict_cardiac_ion_channel_activity(input_data,
                                     prediction_type,
                                     predict_hERG,
                                     predict_Nav,
                                     predict_Cav,
                                     device)
```

- `input_data (str or list)`: The input data for which the discriminative models will process. If the entry is a string, it must be either a SMILES string or a path to a prepared h5 file. If it is a list, it must be a list of SMILES strings.
- `prediction_type (str)`: Either 'regression' or 'classification'. Default is 'regression'.
- `predict_hERG (bool)`: Whether to predict hERG activity. Default is True.
- `predict_Nav (bool)`: Whether to predict NaV1.5 activity. Default is False.
- `predict_Cav (bool)`: Whether to predict CaV1.2 activity. Default is False.
- `device (str)`: The device to use for the inference computations. Must be either 'gpu' or 'cpu'. Default is 'gpu'.

Figure S18. Python function to perform inference with the discriminative models.
